# Tunable PVA–Alginate/Fe_3_O_4_ Ferrogels for AMF-Triggered Drug Release and Magnetothermal Hyperthermia

**DOI:** 10.1021/acsomega.5c13348

**Published:** 2026-05-21

**Authors:** Cihangir Boztepe, Şehadet Çağlayan, Asım Künkül

**Affiliations:** † Department of Biomedical Engineering, Faculty of Engineering, 37520 Inonu University, Malatya 44280, Türkiye; ‡ Department of Chemical Engineering, Faculty of Engineering, Inonu University, Malatya 44280, Türkiye

## Abstract

Next-generation drug delivery technologies represent a cornerstone of modern biomedical research, aiming to improve the efficacy, safety, personalization, and sustainability of cancer therapies. In this context, the development of multifunctional ferrogel systems capable of simultaneously enabling controlled drug release and magnetic field–induced hyperthermia is of significant importance. In this study, poly­(vinyl alcohol)–alginate (PVA–Alg)/Fe_3_O_4_ ferrogels containing 0–2 wt % Fe_3_O_4_ were successfully fabricated via a combined ionic and physical cross-linking strategy. Comprehensive structural, morphological, and magnetic characterizations were performed using FT-IR, XRD, SEM, TEM, and VSM analyses. The results demonstrate that increasing Fe_3_O_4_ content significantly reduced the equilibrium swelling ratio (from 72.55 to 18.34 g water/g polymer) and degradation degree (from 46% to 10.3%), while markedly enhancing magnetic saturation (from 0.044 to 28.10 emu/g). Also, the DOX loading capacity decreased from 124 to 76 mg DOX/g polymer due to increased network compactness and reduced availability of active binding sites. Drug release experiments under AMF revealed that both magnetic field strength and Fe_3_O_4_ loading play a decisive role in regulating release kinetics. The equilibrium drug release values ranged from 62.37 to 95.09 mg DOX/g polymer, accompanied by accelerated release rates under higher magnetic field intensities due to magnetothermal heating effects. Overall, the results indicate that PVA–Alg/Fe_3_O_4_ ferrogels exhibit tunable structural, magnetic, and drug release properties, highlighting their strong potential as dual-functional platforms for controlled drug delivery and magnetic hyperthermia–based cancer therapy.

## Graphical abstract

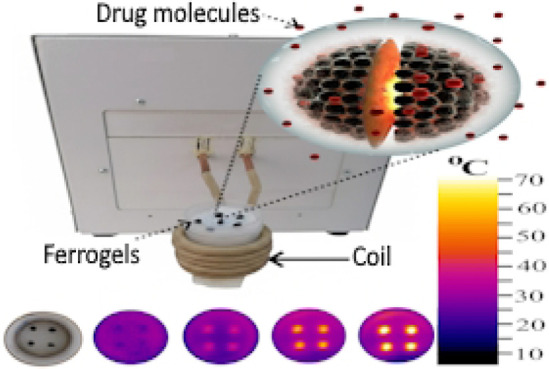

## 1 Introduction

Over the past few decades, hydrogels have emerged as one of the most prominent material classes in biomedical research. These materials consist of three-dimensional, cross-linked networks of hydrophilic polymer chains that are highly swollen yet insoluble in water. Their remarkable swelling capacity, combined with the tunability of their physicochemical properties, has enabled their application in a broad range of biomedical fields, including wound healing, tissue engineering, and controlled drug delivery systems.^1,2^ Key properties of hydrogels, such as biocompatibility, water-holding capacity, and mechanical stability, can be significantly enhanced through appropriate polymer selection and cross-linking strategies.^3^ Poly­(vinyl alcohol) (PVA) is one of the most widely used synthetic polymers for biomedical hydrogel fabrication due to its excellent biocompatibility, tunable mechanical behavior, thermal stability, and biodegradability. Although PVA hydrogels may be cross-linked either chemically or physically, physical cross-linking is often preferred because chemical cross-linkers may introduce cytotoxic residues and alter the chemical structure of the polymer. Among physical cross-linking approaches, the freeze–thaw (FT) method is particularly attractive. PVA hydrogels prepared by repeated FT cycles form crystalline domains that act as physical cross-linking points, thereby improving mechanical strength and structural integrity.^4,5^ The FT technique is simple, environmentally benign, and easily scalable. During the freezing step, ice crystal formation excludes polymer chains into concentrated regions; upon thawing, hydrogen-bonded crystalline microdomains remain intact, generating a physically cross-linked network. Successive FT cycles further reinforce this structure, and typically 3–10 cycles are employed to obtain stable hydrogels. The method provides several advantages, including adequate mechanical robustness, long-term stability at room temperature, and the absence of potentially harmful chemical byproducts.^6,7^

Blending natural polymers with synthetic polymer matrices can markedly enhance the mechanical robustness, biocompatibility, and biodegradability of hydrogel systems. In this regard, sodium alginate is a naturally derived polysaccharide composed of β-*D*-mannuronic (M) and α-*L*-guluronic (G) acid residues, characterized by its high water solubility and remarkable swelling capacity. Alginate chains contain carboxylate groups that readily interact with divalent or multivalent cations to form ionically cross-linked networks, described by the classical “egg-box” model.^8,9^ This type of physical cross-linking, particularly in the presence of Ca^2+^ ions, enhances the structural stability, biocompatibility, and controlled swelling behavior of the resulting hydrogel. In addition, the abundance of carboxylate functionalities in alginate confers a high drug-binding affinity, making alginate-based hybrid hydrogels highly attractive for biomedical and pharmaceutical applications.^10^

PVA–Alg hybrid hydrogels represent a versatile class of multicomponent polymer networks for biomedical applications by integrating the complementary functionalities of both polymers. PVA contributes mechanical robustness, shape retention, and processability through its extensive hydrogen bonding interactions and the formation of semicrystalline domains within the hydrogel matrix.^11,12^ In contrast, sodium alginate, a polyanionic polysaccharide composed of M- and G-block residues, undergoes ionic cross-linking with divalent cations such as Ca^2+^ through the well-defined “egg-box” mechanism, thereby enhancing biocompatibility, swelling control, and structural stability.^13,14^ When combined, the physically cross-linked domains of PVA and the ionically coordinated junction zones of alginate generate a hybrid network with high water content, tunable swelling behavior, and balanced mechanical characteristics. The coexistence of multiple cross-linking modes—hydrogen bonding, crystalline microdomains, and Ca^2+^-mediated ionic bridges—further improves the elasticity, resilience, and long-term stability of PVA–Alg composite hydrogels.^15,16^ However, drug release from conventional PVA–Alg hydrogels is predominantly governed by passive diffusion, meaning that the release kinetics remain largely unresponsive in the absence of external stimuli such as pH variation, temperature change, ionic strength fluctuations, or enzymatic activity. For advanced therapeutic applications requiring controlled, targeted, or stimuli-responsive drug delivery, additional design strategies are necessary. These include the incorporation of functional moieties, the embedding of nanoscale fillers, or the integration of smart polymer segments capable of responding to environmental or externally applied triggers. Such modifications impart dynamic, stimuli-responsive characteristics to the hydrogel system, enabling more precise regulation of drug release profiles and improved therapeutic efficacy.^17,18^

In recent years, natural polymer–based hydrogels have emerged as promising platforms for smart wound dressings and controlled drug delivery systems owing to their excellent biocompatibility and intrinsic bioactivity. In the literature, universal gelation strategies based on electrostatic interactions between lysozyme nanofibers and divalent anions have been reported to effectively inhibit diabetic wound infections through activation of the cGAS–STING signaling pathway.^19^ Moreover, bioenvironment-responsive hydrogel platforms that enable the in situ synthesis of conjugated polymers within the hydrogel matrix are of particular interest, as they allow real-time infection monitoring in combination with photothermal therapy.^20^ Notably, injectable hydrogel systems employing near-infrared (NIR)–responsive conjugated polymers, such as poly­(*N*-phenylglycine), either as backbone components or through supramolecular self-assembly strategies, have demonstrated high photothermal conversion efficiency and “on-demand” drug release capability, thereby generating synergistic therapeutic effects in chemo-photothermal therapy.^21,22^ In addition to these light-triggered systems, the integration of metal oxide nanoparticles, which can respond to external stimuli such as magnetic fields, into polymeric matrices has opened a new dimension in biomedical applications. Furthermore, magnetic stimuli offer a strong alternative and complement to photothermal approaches in terms of deep tissue penetration and remote controllability.

Superparamagnetic Fe_3_O_4_ nanoparticles exhibit field-responsive behavior that enables magnetic guidance, heat generation, and orientation under externally applied static or alternating magnetic fields (AMF).^23^ The incorporation of such nanoparticles into hydrogel matrices has led to the development of magneto-responsive or “smart” hydrogels, commonly referred to as ferrogels. Compared to conventional hydrogels, ferrogels display several advanced functionalities, including stimuli-triggered drug release, magnetothermal hyperthermia, magnetic targeting, and the ability to support combined therapeutic modalities such as chemo-hyperthermia for cancer treatment.^23–25^

Fe_3_O_4_ (magnetite) nanoparticles are particularly attractive for biomedical use owing to their chemical stability, low toxicity, noncarcinogenic nature, and FDA approval for clinical iron supplementation. In contrast, alternative ferromagnetic materials such as Ni or Co, despite their higher magnetic saturation, are avoided in biomedical settings because of significant cytotoxicity and limited biocompatibility.^26–28^ Under AMF exposure, superparamagnetic nanoparticles like Fe_3_O_4_ convert electromagnetic energy into heat primarily through two relaxation mechanisms. (*i) Néel relaxation:* the magnetic moment of each nanoparticle reverses internally to follow the oscillating magnetic field, and the associated energy dissipation manifests as heat. *(ii) Brownian relaxation:* the entire nanoparticle physically rotates within the surrounding fluid to realign with the field direction, generating heat through viscous friction.^29,30^ The combined outcome of these processes results in magnetothermal heating, and the generated heat is rapidly transferred to the surrounding hydrogel matrix. This localized temperature elevation enhances polymer chain mobility, promotes water diffusion, and weakens drug–matrix interactions, thereby facilitating accelerated and magnetically controlled drug release. These magnetothermal and drug-release properties enable ferrogels to function as dual-action therapeutic platforms capable of delivering chemotherapeutic agents while simultaneously inducing localized hyperthermia. Such combined therapy strategies can potentiate cancer cell damage, improve therapeutic efficacy, and offer externally controllable treatment modalities.^31,32^

A survey of the current literature indicates that studies on PVA–alginate or other natural polymer–based ferrogels incorporating Fe_3_O_4_ nanoparticles remain relatively scarce. Although PVA–Alg/Fe_3_O_4_ ferrogel systems have been explored for specific applications—such as antibacterial activity^33^—there is a notable lack of comprehensive investigations addressing both their structural characteristics and their performance in controlled drug release and magnetothermal heating. Moreover, most existing studies employ a single Fe_3_O_4_ concentration, and no systematic work has delineated how variations in nanoparticle loading influence key functional parameters such as swelling behavior, structural stability, AMF-triggered drug release, and magnetothermal conversion efficiency in PVA–alginate-based ferrogels.

These gaps in knowledge hinder the translation of magnetic hydrogel technologies into realistic biomedical applications, where multiple performance criteria—including controlled release kinetics and efficient heat generation—must be simultaneously optimized. To address these shortcomings, the present study synthesized PVA–Alg/Fe_3_O_4_ ferrogels containing graded Fe_3_O_4_ nanoparticle concentrations; conducted detailed structural characterization; evaluated the loading capacity of the model anticancer agent doxorubicin (DOX); systematically quantified magnetothermal heating kinetics and AMF-triggered drug release; and comparatively examined the effects of nanoparticle content on swelling behavior, degradation profile, and DOX release kinetics. This integrated approach enables the assessment of PVA–Alg/Fe_3_O_4_ ferrogels as a dual-functional platform capable of both magnetically controlled drug delivery and localized hyperthermia. By coupling chemotherapeutic action with field-induced heating in a single system, these ferrogels offer important advantages for biomedical applications, including enhanced therapeutic efficacy, spatially selective treatment, externally tunable release behavior, and improved translational potential.

## 2 Materials and Method

Poly­(vinyl alcohol) (PVA, molecular weight 98 000), ferric chloride (FeCl_3_), and doxorubicin hydrochloride (DOX, as model drug) were purchased from Sigma–Aldrich (USA). Alginate (Alg, its sodium alginate form), ammonium hydroxide (NH_4_OH), ferrous sulfate (FeSO_4_), and tannic acid (TA, 99%) were purchased from Sigma–Aldrich (USA) and Merck (Germany). All chemicals were used as obtained, without any additional purification. Deionized water was used in all of the experiments.

### 2.1 Synthesis and Surface Modification of Fe_3_O_4_ Magnetic Nanoparticles (MNPs)

Fe_3_O_4_ magnetic nanoparticles were synthesized via the coprecipitation method. In a 150 mL three-neck round-bottom flask, 100 mL of deionized water was heated to 60 °C under continuous mechanical stirring at 1200 rpm, followed by the addition of 10.8 g FeCl_3_·6H_2_O (0.04 mol) and 5.56 g FeSO_4_·7H_2_O (0.02 mol). After complete dissolution, the reaction mixture was purged with inert N_2_ gas, and a 0.75 M ammonium hydroxide (NH_4_OH) solution was added dropwise (one drop every 5 s) until the pH reached 9, enabling the coprecipitation of Fe_3_O_4_ nanoparticles. Once pH 9 was achieved, the reaction was maintained at 60 °C for an additional 1 h under a nitrogen atmosphere. The mixture was cooled to room temperature and centrifuged at 12 000 rpm for 40 min to collect the Fe_3_O_4_ precipitate. The precipitate was washed three times with deionized water and once with ethanol to remove unreacted ions and impurities. The purified Fe_3_O_4_ nanoparticles were then dried under vacuum at 50 °C for 48 h.

For surface modification, 100 mg of dried Fe_3_O_4_ nanoparticles were dispersed in 20 mL of deionized water using an ultrasonic bath for 30 min. The pH of the suspension was adjusted to 8.0 using Tris buffer. While stirring at 800 rpm, 2 mL of a 5 mg/mL tannic acid (TA) solution was added dropwise, and the mixture was allowed to react for 30 min to facilitate TA adsorption onto the nanoparticle surface. The TA-modified nanoparticles were collected by centrifugation at 12,000 rpm for 15 min, washed three times with deionized water, and dried under vacuum at 50 °C. A 10 mg/mL suspension of the modified Fe_3_O_4_ nanoparticles was prepared by sonicating the particles in deionized water for 1 h, and this stock suspension was used for subsequent experiments.

### 2.2 Preparation of PVA and Alg Solutions

A 14% (w/v) poly­(vinyl alcohol) (PVA) solution was prepared by dissolving 14 g of PVA in 100 mL of deionized water. The mixture was heated to 90 °C in a round-bottom flask equipped with a reflux condenser and stirred using a magnetic stirrer at 900 rpm for 3 h until a clear and homogeneous solution was obtained. A 2% (w/v) sodium alginate (Alg) solution was prepared separately by dissolving the required amount of alginate powder in deionized water under continuous magnetic stirring at 750 rpm at 50 °C for 12 h, ensuring complete hydration and dissolution of the polymer.

### 2.3 Preparation of PVA–Alg/Fe_3_O_4_ Solutions

For the preparation of PVA–Alg/Fe_3_O_4_ precursor solutions, a volumetric ratio of 3:2 (v/v) for PVA to alginate was selected. This ratio was chosen to achieve appropriate viscosity, prevent filament formation during droplet generation, obtain mechanically stable spheres, and ensure high structural stability after the freeze–thaw (F/T) process. Composite solutions containing 0.5, 1.0, 1.5, and 2.0 wt % Fe_3_O_4_ (based on the total mass of PVA and alginate) were prepared. For this purpose, 30 mL of PVA solution was transferred into a 100 mL glass flask and stirred at 900 rpm, while the required volume of surface-modified Fe_3_O_4_ nanoparticle suspension (10 mg/mL) was added dropwise. Subsequently, 20 mL of the alginate solution was added dropwise to each mixture, and stirring was continued for 3 h to ensure homogeneity. To obtain Fe_3_O_4_ contents of 0.5, 1.0, 1.5, and 2.0 wt %, 2.3, 4.6, 6.9, and 9.2 mL of the Fe_3_O_4_ suspension (10 mg/mL) were added, respectively. No Fe_3_O_4_ suspension was added to the control formulation (PVA–Alg without magnetic nanoparticles).

### 2.4 Preparation of PVA–Alg/Fe_3_O_4_ Ferrogels

Ionically cross-linked PVA–Alg/Fe_3_O_4_ ferrogels were produced via the droplet-gelation method using a CaCl_2_ coagulation bath. For this purpose, PVA–Alg/Fe_3_O_4_ precursor solutions containing different amounts of Fe_3_O_4_ nanoparticles were transferred dropwise into a 0.15 M CaCl_2_ solution stirred at 150 rpm. The solutions were dispensed using a syringe from a height of approximately 10 cm, allowing spherical PVA–Alg/Fe_3_O_4_ beads to form immediately upon contact with the Ca^2+^ ions. The resulting ferrogel spheres were allowed to mature in the CaCl_2_ solution for 10 min, collected by filtration, and washed twice with deionized water to remove excess Ca^2+^.

To introduce physical cross-linking within the PVA chains, the ferrogel spheres were subjected to a freeze–thaw (F/T) process. The beads were placed in Petri dishes and frozen at −24 °C for 20 h, followed by thawing at room temperature (≈22 °C) for 3 h. Each freeze–thaw step was considered one cycle, and three consecutive cycles were applied to enhance structural stability. After the final cycle, the ferrogel spheres were washed with deionized water for 12 h, then dried using a lyophilizer (freeze-drying) system. The ferrogels prepared with 0, 0.5, 1.0, 1.5, and 2.0 wt % Fe_3_O_4_ were coded as FG_0.0_, FG_0.5_, FG_1.0_, FG_1.5_, and FG_2.0_, respectively.

### 2.5 Structural Analysis

The structural features of the synthesized ferrogels were examined using Fourier-transform infrared spectroscopy (FT-IR) in the 4000–450 cm^–1^ range, employing a PerkinElmer spectrometer with samples prepared in KBr pellets. The surface topography and microstructural features of the ferrogels were investigated by scanning electron microscopy (SEM; Leo EVO 40xVP). X-ray diffraction (XRD) analysis using a Rigaku RadB-DMAX II diffractometer. The crystalline structures of the ferrogels were examined using X-ray diffraction (XRD) analysis performed on a Rigaku RadB-DMAX II diffractometer. The magnetic behavior of the ferrogel samples was characterized using a vibrating sample magnetometer (VSM) module of the Quantum Design PPMS-9T platform, under applied magnetic fields ranging from 0 to 60 kOe. High-resolution transmission electron microscopy (TEM) (FEI, 120 kV) was employed to visualize the morphology and nanoscale structure of the synthesized Fe_3_O_4_ magnetic nanoparticles.

### 2.6 Swelling Capacities of Ferrogels

The swelling capacity at equilibrium (*S*_eq_) of ferrogels was calculated using eq 1.

1 $$S_{eq}=\frac{m_{eq}‐m_{0}}{m_{0}}$$

In this equation, *m*_eq_ and *m*_0_ are the masses of equilibrium-swollen and dry ferrogels, respectively.

### 2.7 Degradation Studies of PVA–Alg/Fe_3_O_4_ Ferrogels

The degradation kinetics of the PVA–Alg/Fe_3_O_4_ ferrogels containing different Fe_3_O_4_ ratios were systematically investigated. Degradation experiments were performed using freeze-dried ferrogels with known initial dry masses, which were immersed in phosphate-buffered saline (PBS) at 37 °C to simulate physiological conditions. At three-day intervals, the ferrogels were removed from the PBS solution, and their dry masses were recorded. This procedure was continued until the samples reached a constant mass, indicating equilibrium degradation behavior, which occurred after approximately 50 days. The percentage degradation of the ferrogels was calculated using the following equation.

2 $$D\%=\frac{m_{0}‐m_{t}}{m_{0}}\times 100$$

In this equation, *D*% represents the percentage degradation, and *m*_t_ denotes the mass of the ferrogel at time t.

### 2.8 Drug Loading into PVA–Alg/Fe_3_O_4_ Ferrogels

Drug loading was performed using freeze-dried PVA–Alg/Fe_3_O_4_ ferrogels with known dry masses. The ferrogel spheres were immersed in 100 mL of a 200 ppm doxorubicin (DOX) solution and stirred at 450 rpm for 24 h to allow adsorption of DOX onto the hydrogel matrix. Following the loading process, the remaining DOX concentrations in the solutions were quantified using a UV–Vis spectrophotometer, and the drug loading capacity (mg DOX/g dry polymer) was calculated using eq 3:

3 $$Q_{cap}=\frac{C_{0}‐C_{\mathrm{eq}}}{m_{0}}$$

here, *Q*_cap_ is the DOX adsorption capacity of the hydrogel (mg DOX/g dry polymer), *C*_0_ is the initial amount of DOX in solution (mg), and *C*_eq_ is the amount of DOX remaining in solution at equilibrium (mg).

### 2.9 Heating and Drug Release from DOX-Loaded Ferrogels under an Alternating Magnetic Field

Drug release experiments from DOX-loaded PVA–Alg and PVA–Alg/Fe_3_O_4_ ferrogel spheres were performed using a commercial induction heating system (ONX6 Induction), as shown in Figure 5A. The induction system consists of two primary components: a power conversion inverter unit and a water-cooled copper solenoid coil. The system is equipped with a solenoid coil of 34 mm diameter and 5 turns, operating at a maximum power output of 5.5 kW and a working frequency of 360 kHz. For each experiment, a known mass of DOX-loaded ferrogels was placed into a plastic tube containing 50 mL of deionized water, which was then positioned inside the induction coil. Drug release kinetics were investigated under alternating magnetic fields (AMF) with magnetic flux densities of 1.00, 1.25, and 1.50 mT over predetermined time intervals. At specific time points, 0.2 mL aliquots were withdrawn from the release medium, and DOX concentrations were determined via UV–Vis spectrophotometry. Concurrently, temperature variations were recorded in real time using a thermometer inserted through the tube cap. To ensure the accuracy of the magnetothermal measurements and to decouple the inductive heating of the medium from the true magnetothermal response of the nanoparticles, two sets of control experiments were performed. First, 50 mL of deionized water (blank control) was subjected to the maximum AMF conditions to assess the background induction heating. Second, Fe_3_O_4_-free PVA–Alg hydrogels were tested under the same parameters to evaluate the potential heating of the polymer matrix itself.

The specific absorption rate (SAR) of the PVA–Alg/Fe_3_O_4_ ferrogels was calculated to quantitatively evaluate their magnetic heating efficiency under an alternating magnetic field (AMF). SAR values were determined from the initial slope of the temperature–time curves according to the following equation:

4 $$SAR\left(\frac{W}{g}\right)=Cp\cdot \frac{dT}{dt}\cdot \frac{m_{s}}{m_{Fe}}$$

where *C*p is the specific heat capacity of the hydrogel sample (approximated as that of water, 4.184 J/g·K), *dT*/*dt* is the initial slope of the heating curve (K/s), m_s_ is the total mass of the water (*g*), and *m*_Fe_ is the total mass of magnetic iron oxide within the sample (*g*). To ensure measurement accuracy and minimize heat loss, all experiments were conducted within an insulated sample holder to approximate adiabatic conditions.

The amount of drug released at any time *t* was calculated using eq 5.

5 $$q_{t}=\frac{C_{t}}{m_{0}}$$

Here, *q*_t_ represents the cumulative amount of DOX released per gram of dry polymer (mg of DOX/g of polymer), and *C*_t_ is the amount of DOX released into the solution at time t (mg).

The drug release rate was determined using eq 6.

6 $$q_{v}=\frac{q_{t}}{t}$$

with *q*_v_ denoting the release rate (mg DOX/min) and *t* being the release time in minutes. The calculation of *q*_v_ was performed using data collected up to 60 min.

The percentage drug release at equilibrium was calculated using eq 7, defined as

7 $$Q_{eq}=\frac{C_{\max}}{Q_{\mathrm{cap}}}\times 100$$

where *Q*_eq_ is the percentage of DOX released at equilibrium, and *C*_max_ is the amount of DOX released per gram of dry polymer at equilibrium (mg DOX/g polymer).

To determine the on-demand drug release performance of ferrogels in response to an external magnetic stimulus and the reversible nature of their thermal response, an on/off cyclical release test was performed on FG_1.0_ samples. In this experiment, samples were periodically exposed to an AMF of 1.25 mT and 360 kHz frequency. Each cycle included an “on” phase, where the magnetic field was activated to heat the system, followed by an “off” phase, where the AMF effect was allowed to shut off. Temperature changes were recorded in real-time via a thermometer throughout the experiment; cumulative drug release amounts were determined from samples taken at the end of each period. The obtained data were analyzed comparatively with the release profile under continuous AMF exposure to evaluate the pulsatile release characteristics of the system.

### 2.10 Statistical Analysis

All experiments were performed in triplicate (*n* = 3). Results were expressed as means ± standard deviation (SD) with a 95% confidence level.

## 3 Results and Discussions

### 3.1 Design Strategy and Structural Characterization of Ferrogels

The PVA–Alg/Fe_3_O_4_ ferrogels provide a dual mode of action by combining controlled DOX release with AMF-induced hyperthermia, offering a clear therapeutic advantage. While the hydrogel network ensures sustained baseline release, the magnetic nanoparticles enable rapid, on-demand acceleration of drug diffusion when exposed to an alternating magnetic field. This external controllability minimizes systemic exposure and ensures drug delivery only at the target site. At the same time, the localized heating effect of Fe_3_O_4_ enhances apoptotic responses in cancer cells, creating a synergistic chemothermal treatment approach. Such features make the system highly attractive for precision oncology and next-generation drug delivery technologies.^29^ However, to realize this potential in practical applications, optimization of the nanoparticle ratio, polymer matrix, and magnetic excitation conditions is necessary, and these parameters must be systematically investigated.^25,31^ This study aims to fill this gap and make a significant contribution to the literature by presenting a systematic synthesis, characterization, and performance evaluation.

Schematic illustration of the synthesis and fabrication process of PVA–Alg/Fe_3_O_4_ ferrogels is given in Figure 1. The design strategy adopted for the PVA–Alg/Fe_3_O_4_ ferrogel spheres is essentially a multinetwork, multifunctional composite concept, and this is what makes the system powerful for dual use in controlled drug delivery and magnetic hyperthermia. By fixing the PVA:Alg ratio at 3:2 (v/v) and systematically varying the Fe_3_O_4_ content, the study isolates the effect of magnetic nanoparticle loading on network architecture, swelling, drug loading, and AMF-triggered release, without confounding changes in the polymer backbone. PVA contributes a physically cross-linkable, crystallite-forming network under freeze–thaw cycles, while alginate provides Ca^2+^-mediated ionic “egg-box” junctions and carboxylate-rich domains that are highly relevant for drug binding and metal coordination. Similar PVA–alginate interpenetrating networks have been shown to yield mechanically robust, tunable hydrogels when hydrogen bonding, PVA crystallites, and metal–carboxylate coordination act together.^34^ Within this framework, Fe_3_O_4_ nanoparticles are not only passive magnetic fillers but also active structural elements. Surface Fe^2+^ /Fe^3+^ sites can coordinate with PVA–OH and Alg–COO^–^ groups (Fe–O–C, Fe–O–H), effectively behaving as additional physical/ionic cross-linking nodes that tighten the network, decrease mesh size, and reduce free volume available for water and DOX diffusion—an effect widely reported in alginate/PVA–Fe_3_O_4_ hybrid beads and nanocomposite hydrogels.^35^ The use of tannic-acid–modified Fe_3_O_4_ further enriches the interaction palette: TA supplies multiple phenolic groups capable of strong H-bonding with PVA and alginate, while also forming metal–phenolate coordination complexes with Fe on the nanoparticle surface, leading to a thin, adhesive interphase layer. TA–Fe_3_O_4_ and TA-based hydrogels are known to create dense supramolecular networks through combined hydrogen bonding, π–π stacking, and metal–polyphenol coordination, which improve mechanical integrity and control over small-molecule transport.^36^

**Figure 1:**
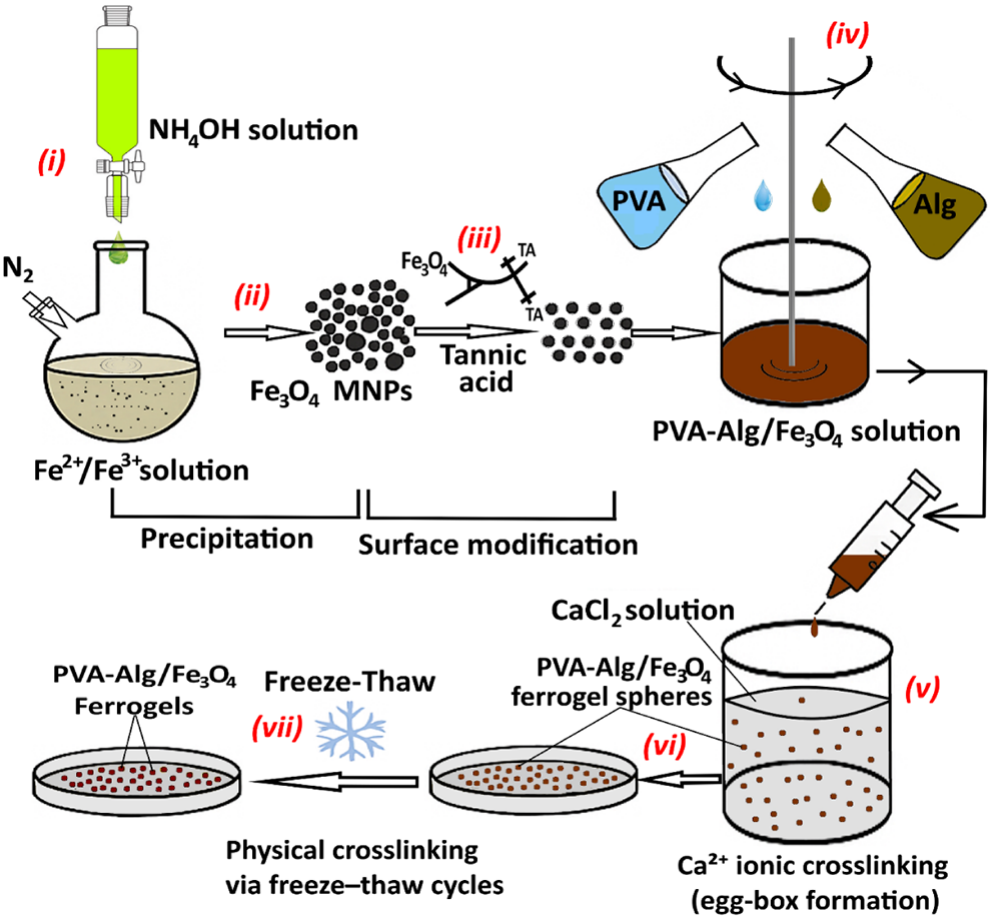
Schematic illustration of the synthesis and fabrication process of PVA–Alg/Fe_3_O_4_ ferrogel spheres. (i) Synthesis of Fe_3_O_4_ MNPs by precipitation method, (ii) Filtration, (iii) Surface modification of MNPs, (iv) Preparation of PVA, alginate, and Fe_3_O_4_ MNPs mixture, (v) Ionic cross-linking with Ca^2+^, (vi) Filtration, (vii) Physical cross-linking via freeze–thaw cycles.

As a result, the ferrogel architecture can be viewed as a hierarchical, trilevel cross-linked system: (i) Ca^2+^–alginate ionic junctions, (ii) PVA crystallites from freeze–thaw cycling, and (iii) Fe_3_O_4_/TA-mediated coordination and hydrogen-bonding bridges. This hierarchy allows independent tuning of mechanical stiffness, swelling ratio, drug loading capacity, and magnetic heating behavior by adjusting Fe_3_O_4_ content and AMF conditions, while keeping the base hydrogel chemistry constant. Magnetic hydrogel reviews emphasize that such rational design—combining a hydrophilic, biocompatible matrix with well-dispersed iron oxide domains—is critical to achieve spatially confined heating and magneto-responsive release, enabling synergistic chemothermal therapy and reducing off-target toxicity.^37^ In this context, the present PVA–Alg/Fe_3_O_4_ design aligns closely with current trends in magnetic hydrogel engineering, where network–nanoparticle interactions are deliberately exploited to couple structural stability, controllable diffusion, and efficient AMF-induced hyperthermia within a single, integrated delivery platform.

The structural integrity of the ferrogels was ensured through a synergistic dual cross-linking approach: physical entanglement via three freeze–thaw cycles for PVA and ionic gelation for the alginate matrix. Although the small spherical geometry (Ø ≈ 2 mm) of the ferrogels poses challenges for conventional macroscale mechanical testing, the literature established for similar hybrid systems confirms that this cross-linking density provides a robust framework suitable for physiological environments. The integration of Fe_3_O_4_ nanoparticles further acts as a reinforcing filler, restricting polymer chain mobility and enhancing the overall network densification, which is consistent with the structural stability observed in our swelling and degradation studies. It was found that Fe_3_O_4_ concentrations above 2.0% compromised the mechanical stability of the ferrogels and led to structural degradation under AMF conditions. Therefore, the optimal threshold to preserve both functional performance and structural integrity was determined to be 2.0%.

FT-IR spectra of PVA, PVA–Alg, and PVA–-Alg/Fe_3_O_4_ ferrogel are shown in Figure 2A. The FT-IR spectrum of pristine PVA shows characteristic bands associated with its hydroxyl-rich polymer backbone. The broad absorption at 3274 cm^–1^ corresponds to −OH stretching vibrations arising from extensive intermolecular and intramolecular hydrogen bonding. The band at 2909 cm^–1^ is attributed to the aliphatic CH_2_ stretching modes, while the peak at 1600 cm^–1^ originates from the bending vibration of residual O–H groups. The signals at 1416 cm^–1^ and 1326 cm^–1^ correspond to CH_2_ and C–H bending vibrations, respectively. A strong band at 1086 cm^–1^ reflects C–O stretching of alcoholic groups, and the peaks at 916 cm^–1^ and 843 cm^–1^ are assigned to C–C and C–H deformation modes typical of the PVA backbone. In the PVA–Alg composite spectrum, the broad absorption near 3256 cm^–1^ corresponds to −OH stretching from both PVA and alginate. The slight shift of this band toward lower wavenumbers compared to pure PVA indicates the formation of stronger hydrogen-bonding interactions between the −OH groups of PVA and the −COO^–^/–OH functionalities of alginate. The CH_2_ stretching mode of PVA shifts to 2914 cm^–1^, further reflecting polymer–polymer interactions. The bands at 1599 cm^–1^ and 1411 cm^–1^ correspond to the asymmetric and symmetric stretching vibrations of the alginate carboxylate groups (COO^–^), confirming the incorporation of alginate into the network. The 1080 cm^–1^ band, assigned to C–O–C and C–O stretching, becomes more pronounced due to the combined contributions of PVA and the glycosidic C–O–C linkages of alginate. The increased intensity of this region suggests substantial overlap between the PVA and alginate backbones. The peak at 819 cm^–1^ corresponds to C–H bending vibrations. For the PVA–Alg–Fe_3_O_4_ composite, significant peak shifts provide direct evidence of polymer–nanoparticle interactions. The −OH stretching band shifts from 3256 cm^–1^ to 3297 cm^–1^, while the alginate COO^–^ bands at 1599 cm^–1^ and 1411 cm^–1^ shift to 1606 cm^–1^ and 1420 cm^–1^, respectively. These changes indicate coordination of hydroxyl and carboxylate groups to Fe centers on the nanoparticle surface. Similarly, the C–O–C/C–O stretching band shifts from 1086 cm^–1^ to 1089 cm^–1^, suggesting Fe–O–C type interactions between PVA/alginate oxygen atoms and surface Fe sites. Collectively, these spectral modifications confirm the formation of a well-defined Fe_3_O_4_–polymer interfacial region and support the presence of ionic and coordinative cross-linking bridges within the composite network.

**Figure 2:**
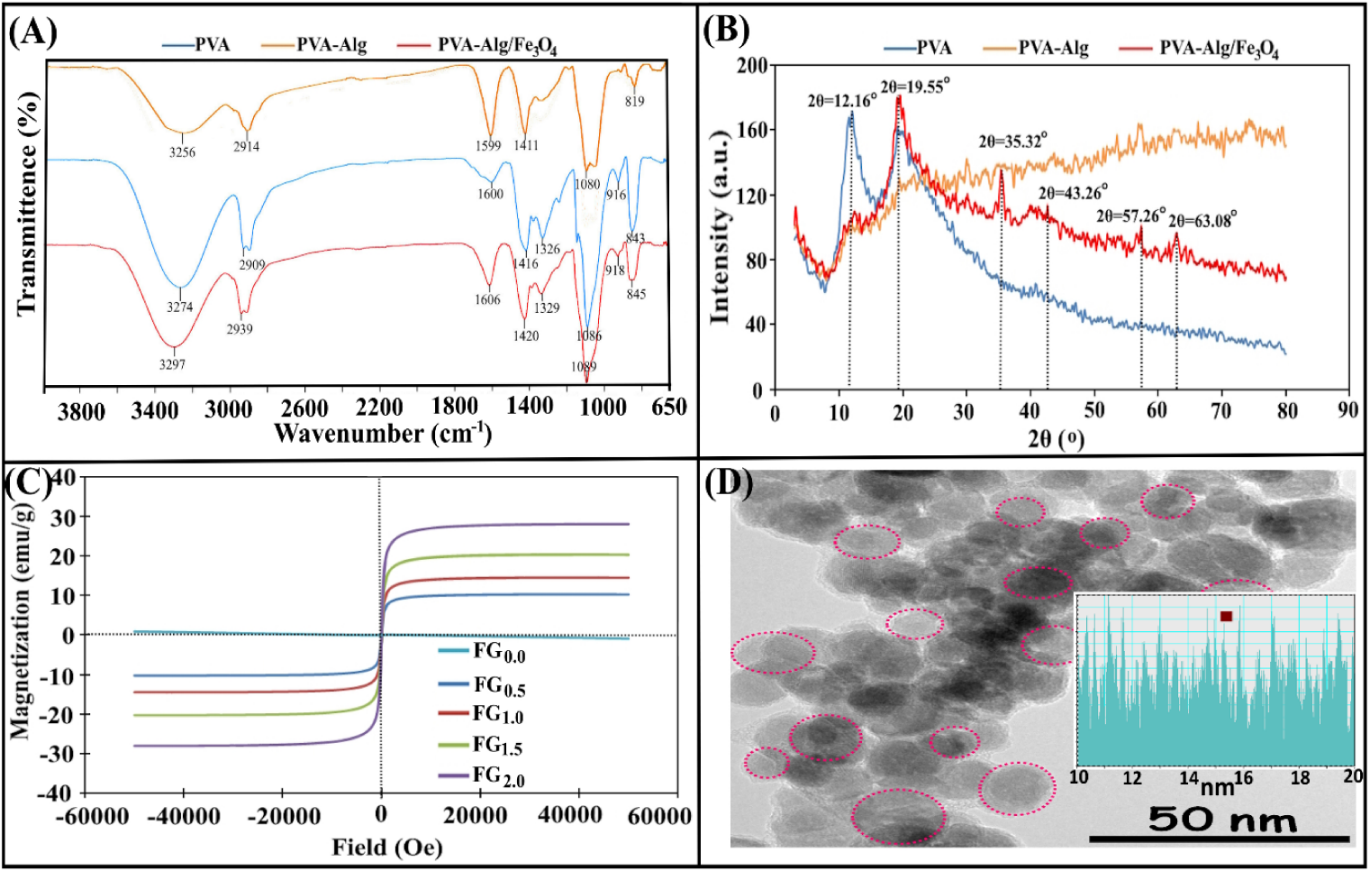
**A)** FT-IR spectra, **B)** XRD spectra of PVA, PVA–Alg, and PVA–Alg/Fe_3_O_4_ ferrogel, **C)** Magnetization curves of PVA–Alg/Fe3O4 ferrogel series, **D)** TEM image and size distribution histogram of Fe_3_O_4_ MNPs.

XRD spectra of PVA, PVA–Alg, and PVA–Alg/Fe3O4 hydrogels are given in Figure 2B. Pure PVA displayed a strong characteristic peak (semicrystalline main peak) at 2θ = 19.55° and an additional characteristic peak at 2θ = 12.16°, respectively. The rapid decrease in intensity after 2θ indicates semicrystalline PVA. When the spectrum of PVA–Alg is examined, the significant weakening and broadening of the 19–20° peak in PVA can be explained by the disruption of PVA chain stacking and decreased crystallinity due to strong H-bonding/ionic interactions between PVA and alginate. Broad amorphous halos are evident in the 10–15° and 20–40° ranges, indicating the formation of a more amorphous composite. In the spectrum of PVA–Alg/Fe_3_O_4_, the introduction of Fe_3_O_4_ into the structure additionally suppressed the crystallinity of the polymer and imparted a crystalline magnetic phase to the composite. Sharp peaks specific to Fe_3_O_4_ are seen as the strongest at 2θ = 35.32°, and the spinel magnetite phase at 2θ = 43.26°, 2θ = 57.26° and 2θ = 63.08°. The incorporation of Fe_3_O_4_ nanoparticles leads to a partial disruption of the long-range ordering of polymer chains, as evidenced by the slight broadening and reduced intensity of the characteristic PVA diffraction peaks. This indicates a decrease in the overall crystallinity of the polymer matrix due to restricted chain mobility induced by nanoparticle–polymer interactions. However, the presence of Fe_3_O_4_ does not eliminate PVA crystalline domains but rather alters their spatial distribution within the composite structure.

The magnetization behavior of the PVA–Alg/Fe_3_O_4_ composite ferrogels with varying Fe_3_O_4_ contents was investigated using vibrating sample magnetometry (VSM), and the corresponding hysteresis loops are presented in Figure 2C. All samples exhibit a typical S-shaped magnetization curve without a pronounced hysteresis loop, which is indicative of paramagnetic/superparamagnetic-like behavior rather than ferromagnetic ordering. The pristine PVA–Alg hydrogel shows a negligible saturation magnetization (HMS) value of 0.044 emu/g, confirming its nonmagnetic nature. Upon incorporation of Fe_3_O_4_ nanoparticles into the hydrogel matrix, a significant enhancement in the magnetic response is observed. The HMS values systematically increase with increasing Fe_3_O_4_ content, reaching 10.25, 14.50, 20.28, and 28.10 emu/g for 0.5, 1.0, 1.5, and 2.0 wt % Fe_3_O_4_ loadings, respectively. This direct correlation between nanoparticle concentration and magnetization demonstrates the efficient dispersion and successful integration of Fe_3_O_4_ within the PVA–Alg network. The nearly linear increase in HMS values suggests that the embedded Fe_3_O_4_ particles retain their intrinsic magnetic characteristics without severe agglomeration or shielding effects from the polymer chains. It is also noteworthy that the coercivity (H_c_) and remanence (M_r_) values of all ferrogel samples are close to zero, supporting the superparamagnetic-like nature of the composites. This feature is particularly advantageous for biomedical applications such as targeted drug delivery, hyperthermia treatment, and magnetic resonance imaging (MRI) contrast enhancement, where a rapid magnetic response under an external field and negligible remanence after field removal are highly desirable. The enhancement of magnetic saturation with increasing Fe_3_O_4_ content can be attributed to the larger number of magnetic dipoles per unit mass, which are aligned along the external magnetic field. Meanwhile, the hydrogel matrix provides a biocompatible and flexible environment, preventing extensive aggregation of Fe_3_O_4_ nanoparticles and thereby maintaining their nanoscale magnetic functionality. The combination of tunable magnetization, hydrophilic polymeric structure, and good processability highlights the potential of PVA–Alg/Fe_3_O_4_ ferrogels as promising candidates for magnetically controlled biomedical devices.

The morphological features of the synthesized Fe_3_O_4_ nanoparticles were examined using transmission electron microscopy (TEM), and the obtained micrograph is shown in Figure 2C. The TEM image reveals that the nanoparticles are nearly spherical in shape and are homogeneously distributed, indicating a successful nucleation and growth process during synthesis. From the size distribution histogram, the average particle size is estimated to be in the range of 10–20 nm. The uniform nanoscale size suggests effective control over the precipitation process. The regions highlighted in the micrograph correspond to densely packed nanoparticle clusters, which can be attributed to magnetic attraction forces leading to the formation of small aggregates. Despite this partial aggregation, the overall morphology demonstrates that the synthesis parameters—such as Fe^2+^ /Fe^3+^ molar ratio (1:2), reaction temperature, and controlled base addition rate—enabled the formation of uniform and monodisperse Fe_3_O_4_ nanoparticles. The smooth contrast variation in the TEM image and the absence of amorphous halos further confirm the crystalline nature of Fe_3_O_4_, consistent with the inverse spinel structure previously reported in the literature.^38,39^ Furthermore, the relatively narrow size distribution and clear particle boundaries indicate that the nanoparticles possess good crystallinity and structural stability, which are desirable characteristics for subsequent incorporation into polymeric matrices such as PVA–Alg hydrogels. The nanoscale dimensions of the particles are expected to enhance their surface area-to-volume ratio, improving both the magnetic responsiveness and interfacial interaction within composite ferrogel systems.

Figure 3 displays SEM micrographs of freeze-dried PVA–Alg/Fe_3_O_4_ ferrogels containing various Fe_3_O_4_ concentrations, revealing the effect of magnetic nanoparticle incorporation on surface morphology and network compactness. The pristine PVA–Alg hydrogel sphere (Figure 3A) exhibits a highly porous, fibrillar structure composed of loosely entangled PVA and alginate chains. The presence of abundant free filaments and irregular pores suggests that the network is mainly governed by physical entanglement and hydrogen bonding between PVA hydroxyl and alginate carboxylate groups. Owing to the absence of inorganic components, the surface appears soft and less compact, characteristic of a weakly cross-linked hydrogel. At low Fe_3_O_4_ loading (0.5 wt %, Figure 3B), localized densification and reduced fibrillar looseness are observed. The sparsely distributed Fe_3_O_4_ nanoparticles act as electrostatic anchoring centers, promoting Fe–O coordination with PVA and alginate functional groups, which partially restricts polymer chain mobility and results in a more compact but still porous structure. Increasing Fe_3_O_4_ content to 1 wt % (Figure 3C) produces a more homogeneous and dense morphology with fewer free PVA segments and better-defined pores. The uniformly dispersed nanoparticles form multiple interfacial coordination bridges (Fe–O–C and Fe–O–H), enhancing structural integrity and generating an optimal balance between porosity and stability. At 1.5 wt % Fe_3_O_4_ (Figure 3D), the surface becomes highly compact with minimal visible fibrils, suggesting effective nanoparticle-mediated bridging of polymer chains and increased cross-linking density. Further increase to 2 wt % Fe_3_O_4_ (Figure 3E) leads to a rigid and densely packed structure with partial nanoparticle aggregation and increased surface roughness, indicating strong inorganic–organic coupling but reduced flexibility. The low-magnification image (Figure 3F) shows that even at 2 wt % Fe_3_O_4_, the ferrogel spheres maintain a uniform spherical shape and smooth surface, confirming that Ca^2+^–Alg ionic cross-linking combined with Fe_3_O_4_-induced secondary interactions ensures structural integrity during freeze-drying. Overall, the progressive addition of Fe_3_O_4_ nanoparticles transforms the PVA–Alg matrix from a loose hydrogel into a compact, magnetically responsive composite. The nanoparticles serve as both magnetic fillers and physical cross-linking nodes, reinforcing the network through hydrogen bonding and electrostatic coordination, thereby improving mechanical robustness and dimensional stability.

**Figure 3:**
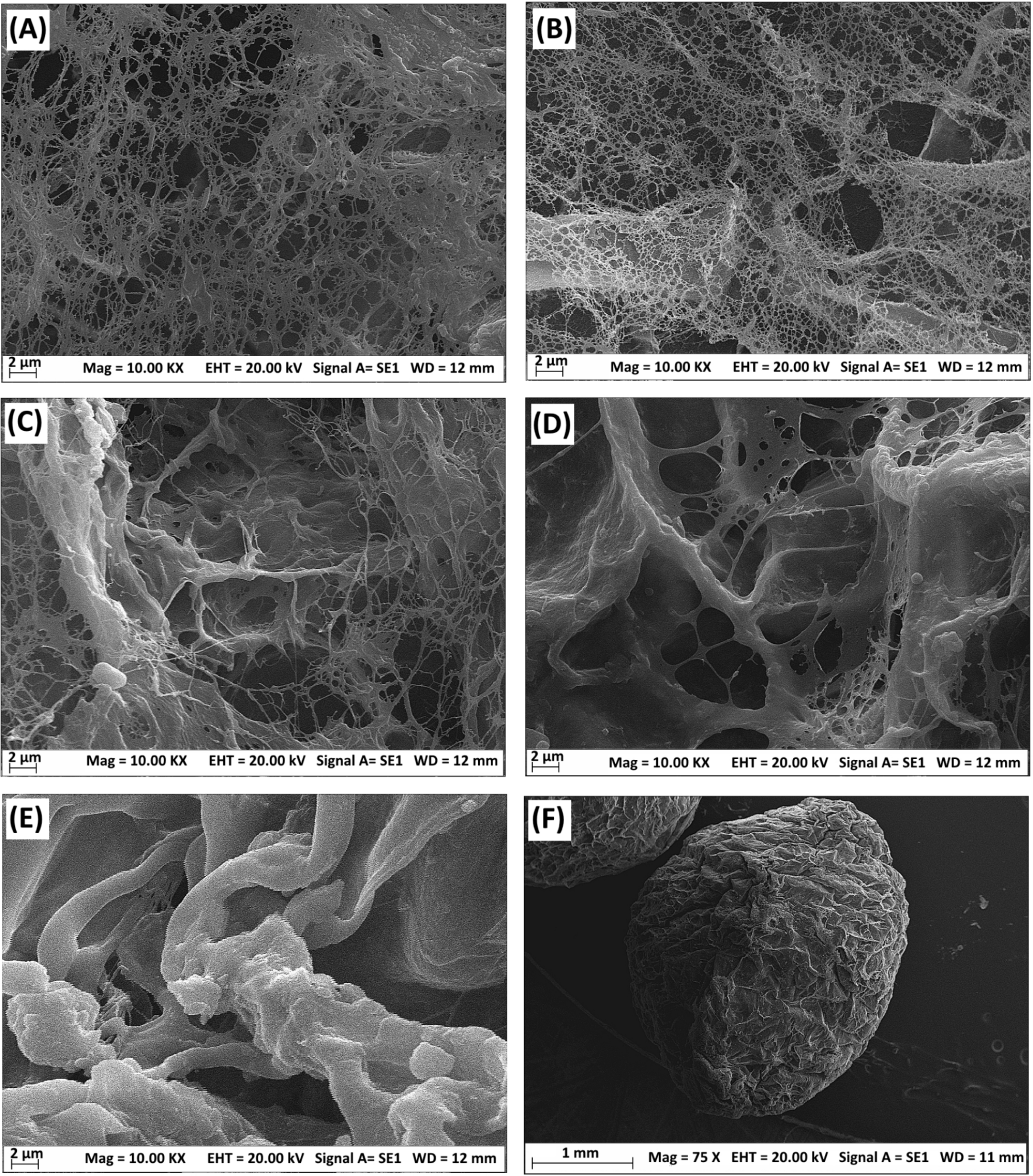
SEM images of the freeze-dried PVA–Alg/Fe_3_O_4_ ferrogels containing various Fe_3_O_4_. (A) PVA–Alg hydrogel (FG_0.0_), (B) 0.5 wt% Fe_3_O_4_-containing PVA–Alg/Fe_3_O_4_ ferrogel (FG_0.5_), (C) 1.0 wt% Fe_3_O_4_ (FG_1.0_), (D) 1.5 wt% Fe_3_O_4_ (FG_1.5_), (E) and (F) 2.0% Fe_3_O_4_ (FG_2.0_). A–E images were acquired at a magnification of × 10,000, and the scale bar corresponds to 2 μm. Image F was acquired at a magnification of × 75, and the scale bar corresponds to 1 mm.

The structural integrity and reproducibility of the magnetothermal performance of the ferrogels are strongly dependent on the spatial distribution of Fe_3_O_4_ nanoparticles within the polymeric matrix. Beyond the structural densification observed in SEM micrographs, elemental mapping analysis focusing on iron (Fe) was performed to verify the homogeneity of nanoparticle distribution throughout the volumetric structure of the spherical ferrogels. From Figure S1, the EDS maps reveal a progressive increase in the intensity of Fe-related signals (displayed in red) with increasing Fe_3_O_4_ content from FG_0.5_ to FG_2.0_. For the FG_0.5_ and FG_1.0_ samples, iron signals are uniformly distributed across the entire cross-sectional area, indicating successful incorporation of magnetic nanoparticles into the three-dimensional PVA–Alg network without the formation of macroscale agglomerates. This homogeneous dispersion suggests effective confinement of Fe_3_O_4_ nanoparticles within the polymer matrix. In the FG_1.5_ and FG_2.0_ samples, localized regions with higher Fe signal intensity are occasionally observed, which can be attributed to partial nanoparticle aggregation at higher loadings. Nevertheless, even at elevated Fe_3_O_4_ contents, the overall matrix maintains a predominantly homogeneous dispersion behavior. Such uniform distribution is essential for achieving spatially balanced heat generation under an applied magnetic field and for maintaining controlled and reproducible drug release kinetics throughout the ferrogel structure.

To further validate macroscopic heating stability with microscopic structural evidence, SEM-EDS elemental mapping was performed on FG_1.0_ ferrogels after 2, 3, 4, and 5 consecutive AMF cycles (Figure S2). Elemental maps for iron (Fe) suggest that the homogeneous spatial distribution of nanoparticles is partially maintained up to the fifth cycle. Partial localized agglomeration due to magnetic dipole–dipole interactions during repeated AMF exposure does not exhibit signs of macroscale agglomeration. These findings provide conclusive evidence that the structural integrity of the ferrogels remains intact, ensuring long-term reliability for multiple-dose therapeutic applications.

### 3.2 Swelling Tests of PVA–Alg/Fe_3_O_4_ Ferrogel Series

The equilibrium swelling values of the ferrogel series are given in Figure 4A. The equilibrium swelling ratio decreased monotonically from 72.55 ± 3.22 g water/g polymer (0% Fe_3_O_4_) to 47.65 ± 2.18, 33.00 ± 1.37, 22.30 ± 1.13, and 18.34 ± 1.03 for 0.5, 1.0, 1.5, and 2.0 wt % Fe_3_O_4_, respectively. This trend is consistent with the expected reduction of network mesh size (ξ) and osmotic driving force as additional physical/ionic cross-links are introduced. In our system, three concurrent mechanisms suppress swelling: *(i) Alginate ionic cross-linking (egg-box)*: During gelation, Ca^2+^ coordinates guluronate blocks, forming junction zones that already limit chain mobility and solvent uptake; stronger ionic coupling generally yields lower swelling in Ca–alginate gels.^38^ *(ii) PVA crystallite cross-linking (freeze–thaw)*: FT processing creates PVA crystallites acting as permanent physical cross-links; higher crystallite density increases the elastic restoring force opposing mixing, thereby decreasing water uptake as predicted by Flory–Rehner concepts.^41^ *(iii) Fe*_*3*_ *O*_*4*_ *–polymer interfacial coordination and charge screening*: Fe_3_O_4_ nanoparticles introduce multivalent surface iron sites that coordinate with alginate −COO^–^ and PVA −OH groups (Fe–O–C/Fe–O–H), functioning as additional cross-linking nodes and restricting chain mobility. Simultaneously, electrostatic interactions between anionic polymer groups and cationic iron centers diminish Donnan osmotic pressure, further limiting solvent influx; increasing Fe_3_O_4_ content strengthens these effects, explaining the stepwise decline in swelling. Analogous Fe/alginate coordination and Fe_3_O_4_–polymer coupling lowering swelling have been widely reported in Fe–alginate and Fe_3_O_4_–polymer hydrogels.^42^

**Figure 4:**
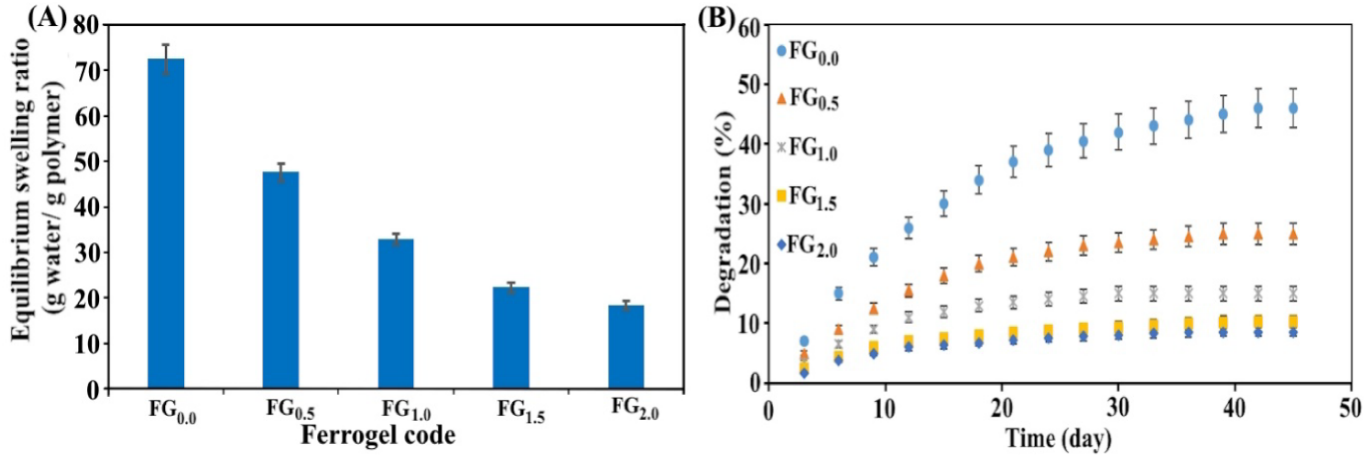
(A) Equilibrium swelling ratios of PVA–Alg/Fe_3_O_4_ ferrogels in distilled water, (B) degradation profiles of ferrogels as a function of time in PBS (pH 7.4) at 37 °C.

Taken together, Ca^2+^–alginate junctions provide the primary ionic network, freeze–thaw PVA crystallites supply a secondary physical network, and Fe_3_O_4_ nanoparticles act as tertiary cross-linking/anchoring centers and local charge screens. The cumulative increase in effective cross-link density and reduction in mobile counterions with Fe_3_O_4_ loading quantitatively accounts for the observed drop in swelling from ∼72.6 to ∼18.3 g/g. This hierarchical cross-linking strategy—ionic (Ca–alginate) + physical (PVA crystallites) + nanoparticle-mediated coordination—produces progressively denser, stiffer, and more dimensionally stable composites at higher Fe_3_O_4_ contents, in line with prior reports on alginate/PVA–Fe_3_O_4_ hybrids.^40–43^

### 3.3 Degradation Behavior of PVA–Alg/Fe_3_O_4_ Ferrogel Series

Degradation experiments conducted in PBS at 37 °C reveal the time-dependent mass loss evolution of the PVA–Alg hydrogel and PVA–Alg/Fe_3_O_4_ ferrogels containing different Fe_3_O_4_ loadings (Figure 4B). Overall, a pronounced decrease in the degradation rate is observed with increasing Fe_3_O_4_ content. This behavior is in good agreement with previously reported ionic dissolution mechanisms and network stabilization effects in alginate- and PVA-based composite hydrogels.^44,45^ The relatively high degradation degree of the Fe_3_O_4_-free PVA–Alg hydrogel (FG_0.0_), reaching approximately 50% mass loss after 50 days, can be attributed to the gradual destabilization of Ca^2+^ -mediated “egg-box” junctions within the alginate network. In PBS medium, Ca^2+^ ions are progressively exchanged with monovalent ions such as Na^+^ and K^+^, leading to the disruption of ionic cross-links and accelerated alginate dissolution. This ion-exchange-driven degradation mechanism of alginate hydrogels in physiological buffers has been extensively documented in the literature.^44,45^ As the Ca^2+^–alginate bridges break down, the ionic stabilization of guluronic acid (G-block) domains diminishes, allowing alginate chains to diffuse out of the hydrogel matrix and resulting in continuous mass loss.^44–46^ Although the physically cross-linked crystalline domains formed in the PVA phase via the freeze–thaw (FT) process prevent complete dissolution of the hydrogel, the removal of nonstabilized alginate segments leads to loosening of the composite network and promotes erosion. Similar degradation behavior has been reported for FT-cross-linked PVA hydrogels, where prolonged exposure to physiological conditions increases chain mobility and induces partial erosion over time.^47^ Consequently, the highest degradation rate observed for the FG_0.0_ sample is an expected outcome and is fully consistent with established degradation mechanisms reported for PVA–alginate hydrogels.

The degradation ratios of the ferrogels containing 0.5 and 1.0 wt % Fe_3_O_4_ were approximately 23% and 15%, respectively, which are significantly lower than that of the Fe_3_O_4_-free hydrogel (FG_0.0_). This behavior can be attributed to the formation of secondary cross-linking nodes within the hydrogel network induced by Fe_3_O_4_ nanoparticles. Previous studies have reported that such nanoparticles establish strong physical and coordinative interactions with both the hydroxyl groups of PVA and the carboxylate (−COO^–^) groups of alginate.^48,49^ These interactions lead to a more compact microstructure, reduced porosity, delayed PBS penetration, and a decreased Ca^2+^ ion exchange rate, thereby effectively suppressing degradation. Consistent with this observation, numerous studies have demonstrated that Fe_3_O_4_ nanoparticles enhance the structural stability of natural polymer-based hydrogels.^50,51^ For ferrogels containing 1.5 and 2.0 wt % Fe_3_O_4_, the degradation values further decrease to approximately 9–11%, clearly indicating that these formulations exhibit the highest structural stability among all samples.

The progressive flattening and plateau formation observed in the degradation curves of all samples after a certain period are consistent with the well-established dual-phase degradation behavior of hydrogel systems.^45,52^ In the initial phase (Phase I), occurring predominantly within the first 10–20 days, rapid displacement of Ca^2+^ ions by monovalent ions present in PBS leads to the dissolution of weakly bound alginate segments, accompanied by slight surface erosion of the PVA-rich domains. This stage is characterized by relatively fast mass loss. In the subsequent phase (Phase II), the degradation process becomes diffusion-controlled, as the remaining network mainly consists of physically cross-linked PVA crystallites and compact regions stabilized by Fe_3_O_4_ nanoparticles. During this phase, swelling is minimal, and the degradation rate is markedly reduced. Similar two-stage degradation profiles have been widely reported for polymeric hydrogel systems under physiological conditions.^45,47,52^

The incorporation of Fe_3_O_4_ nanoparticles into the PVA–Alg hydrogel matrix at increasing concentrations significantly enhances the stability of ionic cross-links, strengthens network compactness, and limits water and ion penetration, thereby effectively suppressing degradation in PBS medium. When SEM and XRD analyses are evaluated together, it becomes evident that the degradation behavior of PVA–Alg and PVA–Alg/Fe_3_O_4_ ferrogels is strongly governed by their microstructural characteristics. SEM images of the Fe_3_O_4_-free PVA–Alg hydrogel reveal a loose, fibrous, and highly porous network that facilitates rapid PBS infiltration and promotes the dissolution of Ca^2+^–alginate ionic cross-links. This observation is consistent with XRD results showing relatively low crystallinity and a high amorphous phase fraction, which favor alginate chain detachment and result in an accelerated degradation rate. In contrast, ferrogels containing Fe_3_O_4_ exhibit a denser, more compact, and homogeneous microstructure in SEM images, indicating that Fe_3_O_4_ nanoparticles act as effective physical fillers and binding centers within the polymer matrix. Although the addition of Fe_3_O_4_ reduces the overall crystallinity of the polymer matrix, the remaining PVA crystalline domains formed through physical cross-linking become relatively more prominent and structurally more significant. These localized crystalline regions act as stable physical linkages, enhancing the composite’s resistance to degradation. Therefore, the XRD properties observed during degradation analysis reflect the functional dominance of these PVA crystalline domains rather than an increase in total crystallinity. Consequently, lower swelling, reduced erosion, and significantly suppressed degradation behavior are observed, particularly in ferrogels with higher Fe_3_O_4_ content. These findings confirm that Fe_3_O_4_ nanoparticles effectively regulate degradation kinetics by reinforcing the microstructural stability of the PVA–Alg hydrogel network. Overall, the obtained results demonstrate that PVA–Alg/Fe_3_O_4_ ferrogels are promising candidates for long-term biomedical applications, including controlled drug delivery systems, tissue engineering scaffolds, and implant coating materials.

### 3.4 Heating Behaviors of PVA–Alg/Fe_3_O_4_ Ferrogel Series

In magnetic hyperthermia and magnetically triggered biomedical systems, the selection of alternating magnetic field (AMF) parameters within clinically accepted safety limits is of critical importance for translational applicability. To evaluate the clinical translational potential of the developed PVA–Alg/Fe_3_O_4_ ferrogels, the applied AMF conditions were analyzed based on the Atkinson–Brezovich safety criterion.^53^ The product of magnetic field strength (***H***) and frequency (***f***), expressed as ***H·f***, is considered a key indicator for clinical safety, as excessive values may induce nonspecific heating in healthy tissues due to eddy current formation. According to the widely accepted Brezovich criterion, the ***H·f*** value should not exceed 4.85 × 10^8^ A·m^–1^ ·s^–1^. Under the AMF conditions employed in this study (*f* = 360 kHz and a maximum magnetic flux density of 1.50 mT), the calculated ***H·f*** value is approximately 4.28 × 10^8^ A·m^–1^ ·s^–1^, which remains below the recommended clinical safety threshold. These parameters are consistent with AMF ranges reported in previous clinical and preclinical magnetic hyperthermia studies, indicating that the selected AMF conditions are safe and suitable for biomedical and translational applications.^53,54^

Figure 5A illustrates the experimental setup employed to investigate the magnetothermal heating behavior and AMF-triggered drug release kinetics of DOX-loaded ferrogels. The system consists of an induction heating unit equipped with an inverter box and a water-cooled copper coil, inside which the drug-loaded ferrogel samples were positioned under ambient conditions. Figure 5B presents a photograph of four swollen ferrogels placed within the coil, together with the corresponding infrared thermal images recorded during exposure to the alternating magnetic field (1.25 mT). As observed from the thermal images, a progressive and homogeneous temperature increase occurs within the ferrogel samples upon AMF application. Quantitative analysis using a thermal camera revealed that the average temperatures of the ferrogels reached approximately 38, 46, 54, 63, and 69 °C after 2, 4, 6, 8, and 10 min of magnetic excitation, respectively. This gradual temperature rise confirms the efficient magnetothermal response of the Fe_3_O_4_-containing ferrogels under AMF, which plays a key role in governing the subsequent drug release behavior.

**Figure 5:**
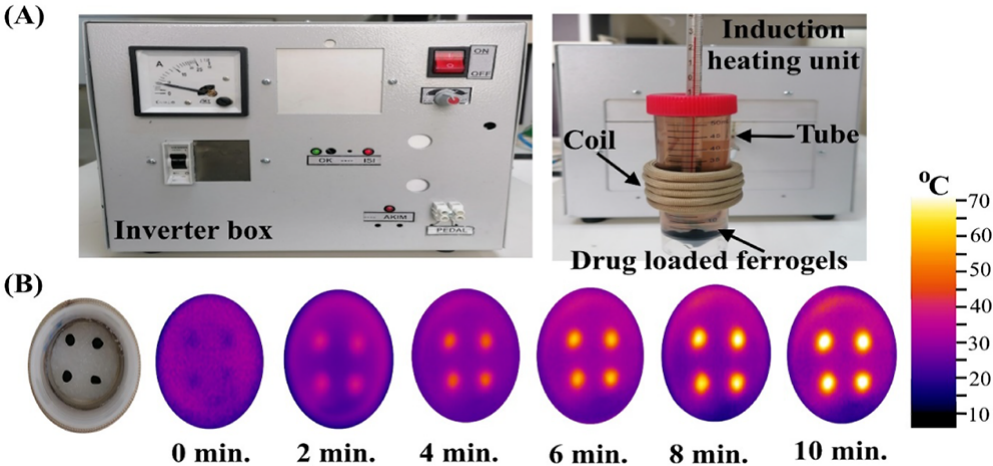
(A) Experimental setup of the induction heating system, (B) photograph and thermal images of the PVA–Alg/Fe_3_O_4_ ferrogels under 1.25 mT AMF heating in a coil (Photographs and thermal images were taken by the authors).

To evaluate the heating efficiency of the synthesized PVA–Alg/Fe_3_O_4_ ferrogels under an alternating magnetic field using a standardized quantitative metric, the specific absorption rate (SAR) was calculated and used for comparison with similar systems reported in the literature. Rather than relying solely on temperature rise profiles, determination of SAR enabled a more precise, mass-normalized assessment of the energy conversion capability of the magnetic nanoparticles within the polymeric matrix. To decouple the potential inductive heating of the aqueous medium or the polymer matrix from the true magnetothermal effect, control experiments were conducted with pure water and Fe_3_O_4_-free PVA–Alg hydrogels. The temperature changes in these blank samples remained negligible (<1 °C) throughout the AMF application period, confirming that the thermal response of the ferrogels is exclusively attributed to the energy dissipation of the embedded magnetic nanoparticles. This analysis was therefore considered a critical parameter for validating the thermal potential of the developed ferrogels with respect to their applicability in magnetic hyperthermia-related biomedical applications.

The SAR values of the PVA–Alg/Fe_3_O_4_ ferrogels, calculated under different AMF strengths, are summarized in Table 1. These values demonstrate that magnetothermal performance is profoundly dictated by both the magnetic field parameters and the nanoparticle loading density. A consistent upward trend in SAR values was observed across all ferrogel groups as the AMF intensity increased. This correlation is fundamentally consistent with the enhanced activation of Néel and Brownian relaxation mechanisms at higher field amplitudes. In established literature, increasing the magnetic field strength is widely reported to amplify the oscillation amplitude of magnetic moments, thereby leading to greater energy dissipation per unit time and subsequently, higher SAR values.^55–57^

**Table 1:** Specific Absorption Rate (SAR) Values of PVA–Alg/Fe_3_O_4_ Ferrogels Under Different AMF Strengths

Ferrogel Code	SAR (W·g^–1^Fe_3_O_4_) values under various AMF		
	1 mT	1.25 mT	1.50 mT
**FG**_**0.5**_	97.15	145.25	232.45
**FG**_**1.0**_	91.7	116.21	159.83
**FG**_**1.5**_	77.50	110.72	146.28
**FG**_**2.0**_	108.95	174.35	203.40

Detailed analysis of the data reveals that the maximum SAR (232.45 W/g) was achieved in the FG_0.5_ sample at 1.50 mT, followed by a decline through FG_1.5_, and a secondary rise in FG_2.0_. At lower loading ratios, the Fe_3_O_4_ nanoparticles are more widely dispersed within the polymer matrix, which minimizes interparticle magnetic dipole–dipole interactions. Since these particles act more as “isolated” entities, their relaxation response to the magnetic field is more efficient, yielding higher heat dissipation per unit mass. Conversely, at moderate to high nanoparticle loadings (FG_1.5_ and FG_2.0_), the limitation in SAR growth can be explained by the intensification of dipole–dipole interactions and the heightened tendency for nanoparticle agglomeration. Such interactions typically create an “energy barrier” that restricts the free rotation of magnetic moments, thus diminishing heating efficiency. These interactions can increase the effective magnetic anisotropy, which prolongs the Néel relaxation time and suppresses a portion of the magnetic losses.^58,59^ Previous studies have similarly reported that optimum SAR values are often attained at moderate concentrations, whereas excessive loading may impair heating efficiency due to these suppressive magnetic interactions.^60,61^ The observed rise in SAR for the FG_2.0_ sample relative to FG_1.5_ may be attributed to a collective magnetic response or shifts in magnetic anisotropy triggered by localized clustering within the matrix. In specific scenarios, controlled clustering can generate “magnetic interaction zones” that paradoxically enhance local heat generation. Furthermore, the increasing density of the polymer network within the ferrogel matrix acts as a critical bottleneck for the Brownian relaxation component. The intensification of both ionic (Ca^2+^–alginate) and physical (PVA crystallites) cross-linking with higher Fe_3_O_4_ content limits the local degrees of freedom for the nanoparticles and imposes viscoelastic constraints. This aligns with literature findings indicating that in rigid and dense network structures, the Brownian contribution is largely suppressed, and SAR values are predominantly governed by Néel relaxation.^62,63^

Overall, the SAR results underscore that magnetic hyperthermia performance in PVA–Alg/Fe_3_O_4_ ferrogels is a complex function not only of Fe_3_O_4_ quantity but also of nanoparticle–polymer interactions, magnetic coupling intensity, and the applied AMF magnitude. This multiparametric behavior suggests that these ferrogel systems offer an optimizable design window for controlled and safe magnetic heating applications, providing SAR values that are quantitatively comparable to similar magnetic hydrogel and ferrogel systems reported in the literature.^57,60,63^

The heating curves shown in Figure 6 illustrate how DOX-loaded PVA–Alg/Fe_3_O_4_ ferrogels, containing different amounts of magnetic nanoparticles, warm up 50 mL of pure water when exposed to several alternating magnetic field (AMF) strengths. Each subplot (A–D) represents ferrogels containing 0.5, 1.0, 1.5, and 2.0 wt % Fe_3_O_4_, respectively, and shows how the temperature of 50 mL of deionized water changes over time when exposed to magnetic flux densities of 1.00, 1.25, and 1.50 mT. When the data are examined together, one can see a rather clear pattern: the more Fe_3_O_4_ incorporated into the hydrogel network, the faster the heating occurs, and the higher the final equilibrium temperature tends to be. For example, the sample containing only 0.5 wt % Fe_3_O_4_ required almost 2 h to reach roughly 30 °C under a 1.00 mT field, whereas the ferrogel with 2.0 wt % Fe_3_O_4_ achieved a much higher steady temperature (around 53 °C) in nearly half that time (∼70 min). This behavior demonstrates the magnetothermal heating capability of the composite system when subjected to AMF. The reason behind the enhanced heating performance at higher Fe_3_O_4_ contents comes from the energy-dissipation mechanisms activated inside magnetic nanoparticles when an alternating field is applied. Superparamagnetic Fe_3_O_4_ particles generate heat mainly through two well-known relaxation processes. In Néel relaxation, the magnetic moment inside each nanoparticle tries to follow the constantly changing field direction; this repeated internal flipping consumes energy, which is eventually released as heat. In Brownian relaxation, the whole nanoparticle physically rotates within the surrounding medium in an attempt to align with the oscillating field, and the frictional resistance during this motion again results in thermal energy generation. The continuous switching of magnetic moments through these two pathways produces magnetic hysteresis losses, and this magnetic energy is transferred to the solution as heat.^29,30^

**Figure 6:**
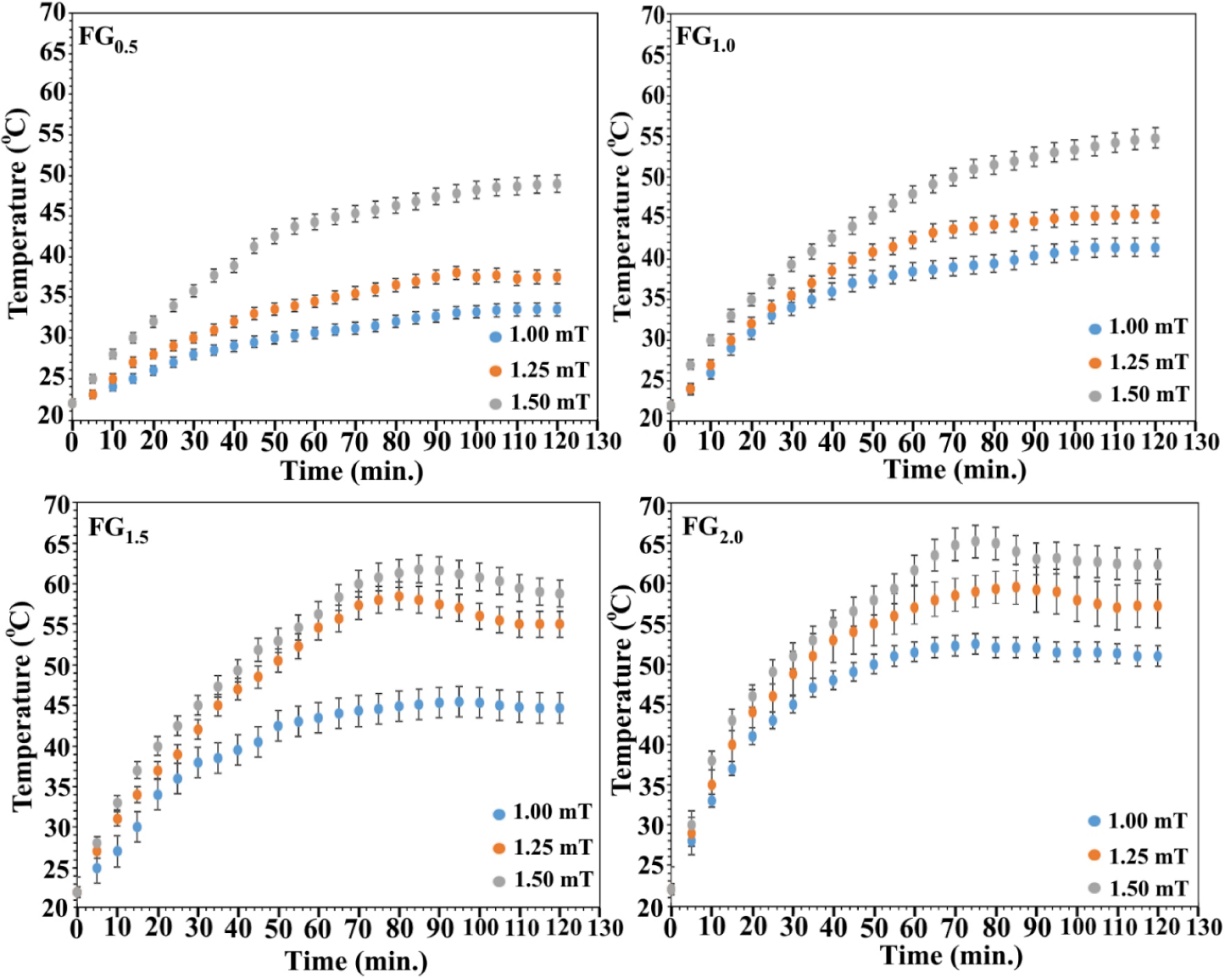
Heating kinetics of PVA–Alg/Fe_3_O_4_ ferrogels with different magnetic nanoparticle loadings exposed to AMFs of 1.00, 1.25, and 1.50 mT.

When the influence of magnetic field strength on the heating profiles is considered, it becomes quite clear that stronger fields tend to push the system toward faster heating and higher final temperatures. This trend mainly stems from the increase in magnetic energy density and the accompanying hysteresis-related losses. In fact, the thermal power generated by magnetic nanoparticles scales roughly with the square of the applied field amplitude (H^2^) as well as the operating frequency. Therefore, as the magnetic field becomes more intense, the amount of energy delivered to the particles also rises, leading to a quicker temperature elevation and a higher thermal equilibrium point. Another factor is associated with hysteresis behavior: at relatively weak fields, the magnetic moments align with the field direction rather easily, but under stronger fields, their reorientation demands more energy. As a result, both Néel and Brown relaxation pathways dissipate more energy, the hysteresis loop effectively broadens, and the overall magnetothermal conversion becomes more efficient.^21,29–31^

A closer look at Figure 6 shows that the ferrogels containing 1.5 and 2.0 wt % Fe_3_O_4_ heat up very quickly under 1.25 and 1.50 mT AMF, reaching relatively high temperatures in the early stage, but then their temperature drops slightly before settling into a steady state. This behavior is commonly seen in systems with high nanoparticle loading, where strong dipole–dipole interactions between particles become more pronounced. In addition, eddy currents (Foucault currents) may develop when the alternating field oscillates at high frequency, producing extra Joule heating and contributing to the rapid initial temperature rise. The combined thermal output from these processes is often described using the SAR, which expresses how much heat (W/g) is generated per unit mass of magnetic nanoparticles.^25,31^ However, at elevated Fe_3_O_4_ contents and higher temperatures, increased dipolar interactions can also promote partial aggregation of nanoparticles. Such clustering tends to reduce the effective surface area and weakens their interaction with the surrounding polymer network, which in turn lowers the overall magnetothermal efficiency of the system. For this reason, the temperature peak followed by a slight decrease—and finally a stable plateau—can be interpreted as a balance between several competing processes: reduced magnetic relaxation efficiency at higher temperatures, enhanced heat dissipation, and the natural tendency of nanoparticles to interact or cluster at high concentrations. Eventually, the system reaches a thermal equilibrium point where heat production and heat loss become equal.

In magnetic hyperthermia-based platforms, investigating the impact of recurrent AMF exposure on the dispersion state of nanoparticles—and consequently on the heating efficiency—is vital for determining long-term durability and reusability. To evaluate the structural and magnetothermal robustness of the synthesized platforms, the FG_1.0_ ferrogel specimens were subjected to five consecutive heating cycles in 50 mL of deionized water under a field intensity of 1.25 mT. The graph relating to heating kinetics is given in Figure S3. The experimental data reveal that the maximum temperatures attained and the initial heating rates remained remarkably consistent across all five cycles. This stability confirms that the Fe_3_O_4_ nanoparticles are securely immobilized within the PVA–Alg hybrid framework, effectively preventing irreversible magnetic-field-induced agglomeration. The high degree of reproducibility in the heating profiles suggests that the system’s specific loss power (SLP) is preserved, indicating that the energy transduction capacity from magnetic to thermal remains intact even after prolonged or repeated stimulation.

Furthermore, the lack of significant performance attenuation by the final cycle suggests that the polymeric architecture is resilient to localized thermal stresses generated during hyperthermia. This also implies that there is no detectable leaching of the magnetic phase from the hydrogel matrix. Ultimately, these cyclical tests validate that the PVA–-Alg/Fe_3_O_4_ ferrogels possess superior thermal and magnetic stability, offering a reliable and clinically sustainable “on-demand” platform for hyperthermia and controlled drug delivery without performance degradation.

### 3.5 Drug Release Behaviors of PVA–Alg/Fe_3_O_4_ Ferrogel Series

In the present work, deionized water was deliberately employed as the release medium in order to decouple magnetothermal effects from ionic contributions originating from the surrounding environment. It is well established that alginate-based networks exhibit pronounced sensitivity to ionic species, where mono- or divalent ions may induce ion-exchange processes that modify cross-linking density, swelling behavior, and matrix integrity.^64^ The use of an ion-free medium, therefore, enabled the assessment of AMF-induced local heating effects on polymer chain mobility and diffusion-driven drug transport without the confounding influence of ion-mediated structural rearrangements. In this context, the adopted experimental design provides a fundamental reference state, allowing the intrinsic “triggered” release behavior of the ferrogels to be evaluated prior to their investigation under more complex, physiologically relevant conditions.

The DOX-loading experiments revealed that the loading capacities of FG_0.0_, FG_0.5_, FG_1.0_, FG_1.5_, and FG_2.0_ ferrogels were 124 ± 3.61, 112 ± 3.09, 101 ± 3.81, 92 ± 2.74, and 76 ± 2.62 mg DOX/g polymer, respectively. The systematic decrease in loading capacity with increasing Fe_3_O_4_ content can be attributed to physicochemical modifications in the hydrogel network induced by the magnetic nanoparticles. Three primary mechanisms are responsible for this behavior: (i) Fe_3_O_4_ nanoparticles function as additional cross-linking nodes through the formation of hydrogen bonds (Fe–O–H) and ionic/coordination bonds (Fe–O–C) with PVA hydroxyls and alginate carboxylates.^65^ These nanoparticle-mediated interactions significantly increase the overall cross-linking density, producing a tighter, more compact network with reduced pore size. Consequently, the diffusion of DOX molecules into the hydrogel interior becomes more restricted. (ii) The functional groups of PVA (−OH) and alginate (−COO^–^) are simultaneously responsible for both drug binding and nanoparticle coordination. As Fe_3_O_4_ content increases, these groups preferentially interact with the Fe^2+^ /Fe^3+^ sites on the nanoparticle surface rather than binding DOX. This competitive occupation of active binding sites reduces the number of available negatively charged regions capable of interacting with the cationic DOX molecules, leading to a progressive decline in drug-loading efficiency.^66^ (iii) Increasing Fe_3_O_4_ content partially neutralizes or shifts the surface charge of the hydrogel toward a less negative (or slightly positive) state. Since DOX·HCl assumes a cationic character at physiological pH, electrostatic repulsion arises between the positively charged nanoparticle-rich regions and DOX molecules. This repulsion further suppresses DOX sorption. Collectively, these effects explain the observed decrease in DOX loading capacity with increasing Fe_3_O_4_ incorporation.^31,67,68^

Figure 7 presents the magneto-responsive DOX release profiles of ferrogels containing different Fe_3_O_4_ contents under magnetic fields of 1.00, 1.25, and 1.50 mT, while Table 2 summarizes the associated release parameters, including the equilibrium release amount (*q*_d_), release rate (*q*_v_), and percentage release (*Q*_d_). The results show that both Fe_3_O_4_ content and magnetic field strength exert pronounced effects on the release behavior. The kinetic curves demonstrate a clear increase in both *q*_d_ and *q*_v_ values with increasing magnetic field intensity. For example, FG_0.5_ exhibited a release rate (*q*_v_) of 0.68 mg DOX/g polymer·min and an equilibrium release (*q*_d_) of 62.37 ± 1.71 mg DOX/g polymer under 1.00 mT, whereas at 1.50 mT, the corresponding values increased to 0.90 mg/g·min and 91.56 ± 1.96 mg/g, respectively. This enhancement is attributed to the direct relationship between heating rate and drug desorption kinetics. Due to the weak and reversible nature of physical interactions between the ferrogel matrix and DOX molecules, the local temperature rise produced by magnetic excitation increases molecular mobility, disrupts drug–matrix interactions, decreases solution viscosity and surface tension within the pores, and ultimately facilitates faster diffusion into the release medium.

**Table 2:** Drug Release Characteristics of FG Ferrogel Series Under AMFs

	*q*_d_ (mg DOX/g polymer)			*q*_v_ (mg DOX/g polymer. min.)			*Q*_d_ (%)		
Sample	Magnetic field strength (mT)								
	*1.00*	*1.25*	*1.50*	*1.00*	*1.25*	*1.50*	*1.00*	*1.25*	*1.50*
**FG**_**0.5**_	62.37 ± 1.71	72.65 ± 1.82	91.56 ± 1.96	0.68	0.82	0.90	55.7 ± 1.15	64.9 ± 1.97	81.8 ± 2.56
**FG**_**1.0**_	83.44 ± 2.03	88.13 ± 1.93	95.09 ± 1.91	0.98	1.05	1.14	82.6 ± 1.33	87.2 ± 2.21	94.1 ± 2.34
**FG**_**1.5**_	79.63 ± 1.97	80.89 ± 2.14	82.55 ± 1.97	0.90	1.01	1.15	86.5 ± 1.46	87.9 ± 2.33	89.7 ± 2.36
**FG**_**2.0**_	66.39 ± 1.74	67.5 ± 1.86	68.74 ± 1.74	0.80	0.90	1.05	85.7 ± 1.49	87.2 ± 2.19	90.4 ± 2.47

**Figure 7:**
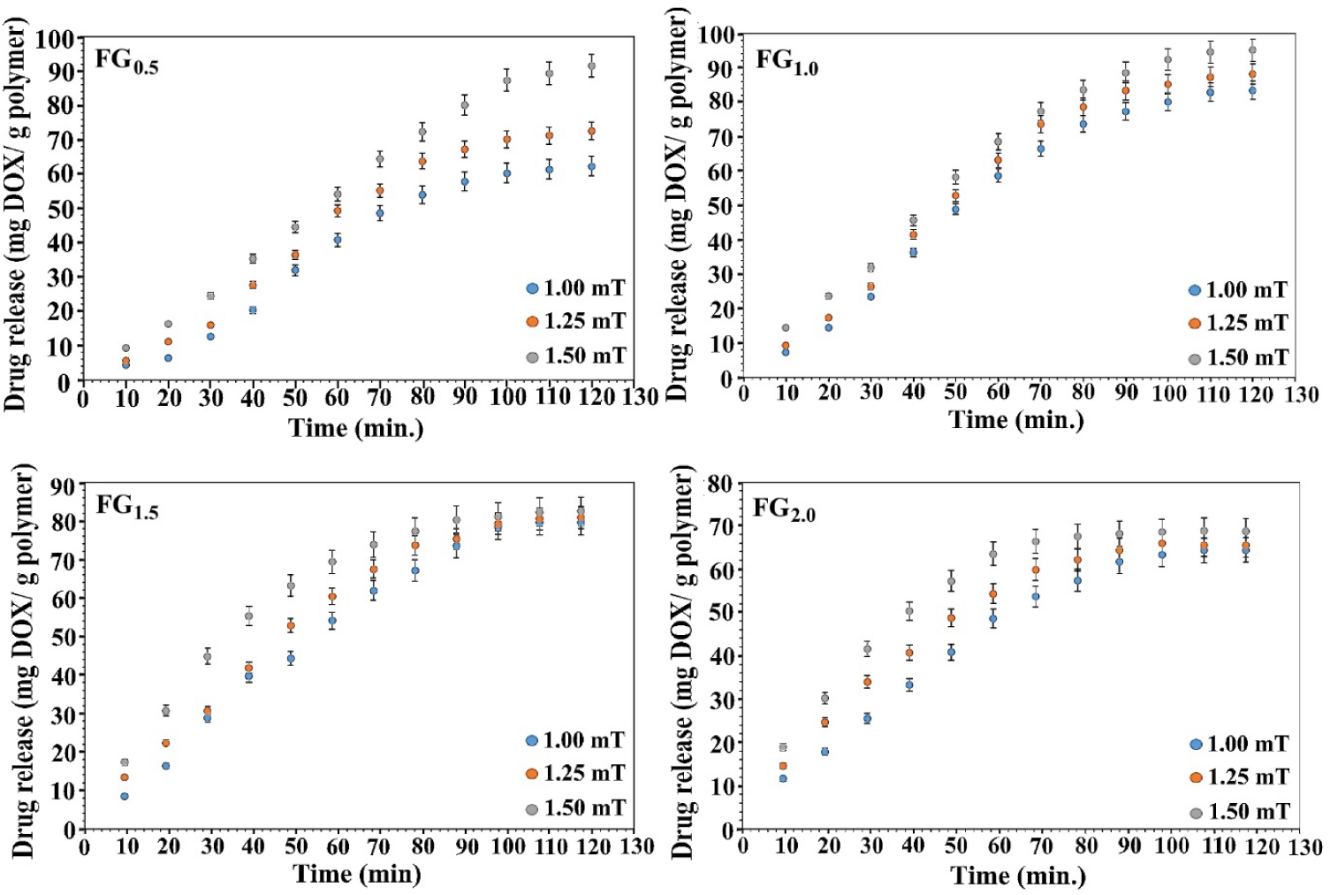
DOX release kinetics of PVA–Alg/Fe_3_O_4_ ferrogels with different magnetic nanoparticle loadings exposed to AMFs of 1.00, 1.25, and 1.50 mT.

At a fixed magnetic field strength, *q*_d_ and *q*_v_ values generally increase with Fe_3_O_4_ content up to 1.0 wt %, after which both parameters begin to decline. For instance, under 1.25 mT, the *q*_d_ values for FG_0.5_, FG_1.0_, FG_1.5_, and FG_2.0_ increase from 72.65 ± 1.82 to 88.13 ± 1.93 mg/g, followed by a decrease to 80.89 ± 2.14 and 67.50 ± 1.86 mg/g, respectively. A similar trend is observed for *q*_v_, increasing from 0.82 to 1.05 mg/g·min, then decreasing to 1.01 and 0.90 mg/g·min. This behavior results from the combined influence of drug loading capacity and magnetic heating efficiency. Although FG_0.5_ possesses a higher DOX loading capacity than FG_1.0_, FG_1.0_ exhibits a higher heating rate and reaches higher temperatures, which enhances drug release; thus, FG_1.0_ shows superior *q*_d_ and *q*_v_ values. Conversely, FG_1.5_ and FG_2.0_ exhibit slightly higher heating rates compared to FG_1.0_ but have significantly lower drug-loading capacities. Therefore, their overall *q*_d_ and *q*_v_ values decrease, indicating that insufficient drug reservoir volume limits the maximum achievable release, despite favorable heating. In addition to the reduced drug loading capacity, the decreased drug release observed at higher Fe_3_O_4_ contents (1.5–2.0 wt %) can be attributed to structural changes in the hydrogel network. The incorporation of higher amounts of Fe_3_O_4_ increases the effective cross-link density and reduces the swelling ratio, which indirectly indicates a decrease in pore size and a more compact polymeric network. Such densification likely restricts the diffusion pathways of drug molecules, leading to slower release kinetics. Although quantitative pore size distribution analysis was not performed, the combined swelling behavior and SEM observations strongly support this diffusion-limited release mechanism.

Analysis of *Q*_d_ (%) values at different magnetic field strengths reveals that the percentage of DOX released increases with the Fe_3_O_4_ incorporation for most ferrogel formulations. This trend is also attributed to the higher temperatures attainable in Fe_3_O_4_-rich systems under magnetic stimulation, which enhance molecular mobility and accelerate release kinetics.

To elucidate the underlying mass transfer physics of DOX from the synthesized PVA–Alg/Fe_3_O_4_ ferrogels, the experimental release profiles were systematically fitted to several mathematical frameworks, including Zero-order, First-order, Higuchi, and Korsmeyer–Peppas models.^53^ The equations for these models are given in Table S1. As summarized by the regression coefficients (*R* ^2^) in Table S2, the Korsmeyer–Peppas model emerged as the most robust fit, yielding *R* ^2^ values consistently exceeding 0.99 across all formulations and AMF intensities. The linear fit visualizations provided in Figure S4 further validate the high fidelity of this model to our empirical data, suggesting that the release process is governed by a complex interplay of polymeric dynamics rather than a singular, elementary mechanism. The specific kinetic parameters (*n* and *k*) derived from the Korsmeyer–Peppas equation (Table S3) offer profound insights into the nature of the transport phenomena. The release exponent (*n*), a diagnostic value for the dominant transport mode in spherical matrices, was analyzed to differentiate between diffusion-led and relaxation-driven processes.^69^ Under 1.00 mT and 1.25 mT fields, the *n* values fluctuated between 0.85 and 1.20 (Table S3). For spherical geometries, values in this range are indicative of “Super Case II Transport.” This confirms that drug mobilization is not merely a concentration-gradient-driven diffusion process but is significantly propelled by the relaxation and swelling of the polymer chains triggered by the AMF. As the field strength was escalated to 1.50 mT, a subtle shift toward lower *n* values (around 0.84) was observed, particularly for the FG_1.0_ and FG_1.5_ groups. This transition signifies that at higher thermal energies, the mechanism shifts toward Anomalous Transport, where Fickian diffusion and polymer chain relaxation contribute more equally to the overall release rate.^70^

The rate constant, *k*, demonstrated a dramatic increase corresponding to the AMF intensity. For instance, in the FG_1.0_ sample, the *k* value surged from 5.39 at 1.00 mT to 18.61 at 1.50 mT. This steep rise quantifies the “on-demand” nature of the system, confirming that the localized heat generated by Fe_3_O_4_ nanoparticles effectively lowers the energy barrier for drug escape by increasing the local chain mobility. In conclusion, the kinetic modeling underscores that the PVA–Alg/Fe_3_O_4_ ferrogels provide an exceptional degree of remote control over drug delivery. The synergy between the structural response of the hybrid matrix and the magnetothermal effect enables the modulation of release kinetics through external AMF parameters. These observations are in excellent agreement with the mechanistic behaviors reported for similar intelligent hydrogel architectures in the literature.^53,69,70^

A paramount advantage of magnetically responsive systems is their capacity for “on-demand” and reversible modulation of therapeutic release profiles via external stimuli. To demonstrate this dynamic control, FG_1.0_ ferrogels were subjected to an AMF on/off cycling protocol at an intensity of 1.25 mT. The resulting temperature and drug release kinetics are presented in Figure S5. Analysis of the thermometric data reveals that the system undergoes rapid localized heating immediately upon AMF activation (“on” state), followed by a cessation of heat generation when the field is deactivated (“off” state). These periodic thermal fluctuations confirm that magnetic hyperthermia provides instantaneous and reversible thermal regulation over the ferrogel platform. The drug release profiles exhibit a high degree of correlation with the thermal transitions, characterized by a distinct “pulsatile” release behavior. During each active AMF cycle, the release rate (represented by the slope) increases substantially; conversely, the rate reverts to a basal level once the magnetic stimulus is withdrawn. This phenomenon substantiates the “gate-like” mechanism, where the generated thermal energy temporarily enhances polymer chain mobility, thereby accelerating the diffusion of DOX through the matrix.

When comparing continuous AMF exposure to the cycling regimen, it is evident that the on/off approach allows for tighter control over cumulative release, minimizing the risk of “burst” overdosing and providing a more sustainable therapeutic window. Consequently, these cycling data unequivocally prove that the developed PVA–Alg/Fe_3_O_4_ ferrogels function not merely as passive carriers but as intelligent, externally commanded delivery platforms. Such dynamic control capabilities indicate a significant clinical translational potential, particularly for chronic treatments requiring patient-specific, precision dosage adjustments. While DOX was utilized as a model agent in this study, the presence of abundant carboxyl and hydroxyl groups within the PVA–Alg matrix provides versatile binding sites for various therapeutics, including metallic drugs like cisplatin.^71^ The AMF-triggered release mechanism, driven by the thermal relaxation of the polymer chains, suggests that this ferrogel platform can be adapted for the controlled delivery of diverse pharmacological agents beyond conventional organic molecules.^72^ These types of magnetothermal-triggered systems can be considered potential carriers not only for local chemotherapy but also for advanced immunotherapy applications by triggering immunogenic cell death (ICD).^73^

## 4 Conclusion

This study was motivated by the need to develop a multifunctional platform capable of integrating controlled drug delivery with magnetic field–induced hyperthermia within a single material system. In this context, PVA–Alg/Fe_3_O_4_ ferrogel spheres containing different Fe_3_O_4_ nanoparticle loadings were successfully fabricated and systematically evaluated. The inherent limitations of conventional PVA–alginate hydrogels, which predominantly rely on passive diffusion and exhibit restricted functional responsiveness, were effectively addressed through the incorporation of magnetic nanoparticles. The experimental findings demonstrate that Fe_3_O_4_ nanoparticles serve a dual role within the hydrogel network, acting not only as magnetic agents but also as secondary physical and coordinative cross-linking centers. Increasing Fe_3_O_4_ content resulted in a pronounced reduction in swelling capacity and degradation rate, indicating the formation of a denser and more structurally stable network. Conversely, the progressive enhancement of magnetic saturation confirmed the ability of the ferrogels to generate an efficient magnetothermal response under alternating magnetic fields (AMF). Drug loading and release investigations further highlighted the dual-functional character of the system. Although higher Fe_3_O_4_ loadings led to reduced drug loading capacity due to network densification, AMF-induced magnetothermal heating significantly accelerated drug release kinetics. Among the studied formulations, ferrogels containing 1.0 wt % Fe_3_O_4_ exhibited the most favorable balance between drug loading efficiency and magnetic heating performance, emphasizing that nanoparticle concentration must be optimized with respect to overall functional performance rather than magnetic response alone.

Overall, this work demonstrates that the structural, magnetic, and release properties of PVA–Alg/Fe_3_O_4_ ferrogels can be finely tuned through controlled Fe_3_O_4_ incorporation. The developed ferrogel systems emerge as promising candidates for AMF-regulated drug delivery combined with localized hyperthermia, offering considerable potential for precision oncology and next-generation stimulus-responsive drug delivery technologies. Furthermore, the findings presented herein provide a robust scientific framework for future in vitro and in vivo investigations.

## Supplementary Material



## Notes

SEM mapping images of the freeze-dried PVA–Alg/Fe_3_O_4_ ferrogels showing the distribution of various Fe_3_O_4_ concentrations (Figure S1). SEM-EDS elemental mapping of iron (Fe) distribution in FG_1.0_ ferrogels after the second, third, fourth, and fifth consecutive AMF heating cycles, confirming long-term structural integrity (Figure S2). Temperature–time profiles of FG_1.0_ ferrogels over five consecutive heating cycles under a 1.25 mT field intensity, demonstrating functional consistency (Figure S3). Mathematical equations for Zero-order, First-order, Higuchi, and Korsmeyer–Peppas kinetic models (Table S1). Correlation coefficients (*R* ^2^) for drug release models under 1.25 mT (Table S2) and detailed Korsmeyer–Peppas model fitting parameters (*n* and *K*) under different magnetic field intensities (Table S3, Figure S4). Finally, the remote-controlled performance of the system is demonstrated through real-time monitoring of FG_1.0_ ferrogels, including temperature–time profiles and cumulative DOX release kinetics under both continuous and on/off cycling AMF exposure at 1.25 mT and 360 kHz (Figure S5) (PDF)
