# Supplementary material for: Tunable PVA–Alginate/Fe3O4 Ferrogels for AMF-Triggered Drug Release and Magnetothermal Hyperthermia
Source: ACS Omega. 2026 May 21;11(21):30841–57. doi: 10.1021/acsomega.5c13348 (PMC13234658; doi:10.1021/acsomega.5c13348)
Supplement: Supplementary file 1 [file ao5c13348_si_001.pdf]

**Supporting Information**

# Tunable PVA–Alginate/$Fe_3O_4$ Ferrogels for AMF-Triggered Drug Release and Magnetothermal Hyperthermia

**Cihangir Boztepe[1*], Şehadet Çağlayan[2], Asım Künkül[2]**

*[1]Department of Biomedical Engineering, Faculty of Engineering, Inonu University, 44280 Malatya, Türkiye*

*[2] Department of Chemical Engineering, Faculty of Engineering, Inonu University, 44280 Malatya, Türkiye*

*Corresponding author: Cihangir Boztepe

E-mail address: cihangir.boztepe@inonu.edu.tr

Telephone : +90 422 377 4715

Fax : +90 422 377 4770

## Contents

**Figure S1.** SEM mapping image of the freeze-dried PVA–Alg/$Fe_3O_4$ ferrogels containing various $Fe_3O_4$. S3

**Figure S2.** Structural stability of $FG_{1.0}$ ferrogels after repeated AMF exposure: SEM-EDS elemental mapping of Iron (Fe) distribution following the 2nd, 3rd, 4th, and 5th heating cycles. S3

**Figure S3.** Magnetothermal stability and reusability of $FG_{1.0}$ ferrogels: Temperature-time profiles over five consecutive AMF heating cycles (1.25 mT) in 50 mL of deionized water. S4

**Table S1.** Different kinetic model equations for drug release S4

**Table S2.** Correlation coefficient (R2) values of drug release kinetics of ferrogels according to different models under 1.25 mT. S4

**Figure S4.** Korsmeyer–Peppas model fitting to ferrogel drug release kinetics S5

**Table S3.** Drug release characteristics of ferrogels for the Korsmeyer–Peppas model S5

**Figure S5.** Real-time monitoring of the remote-controlled behavior of $FG_{1.0}$ ferrogels: (a) Temperature-time profiles and (b) cumulative DOX release kinetics under continuous and on/off cycling AMF exposure (1.25 mT, 360 kHz). S5

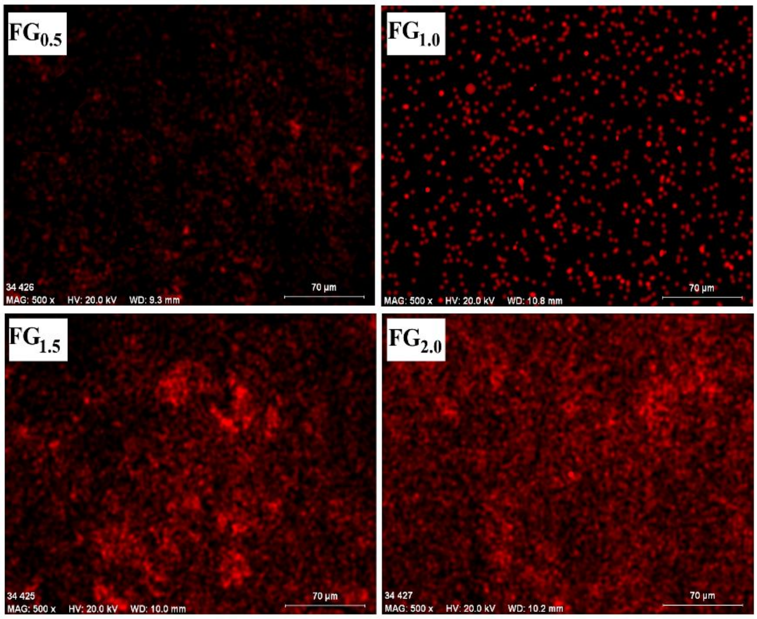


**Figure S1.** SEM mapping image of the freeze-dried PVA–Alg/$Fe_3O_4$ ferrogels containing various $Fe_3O_4$.

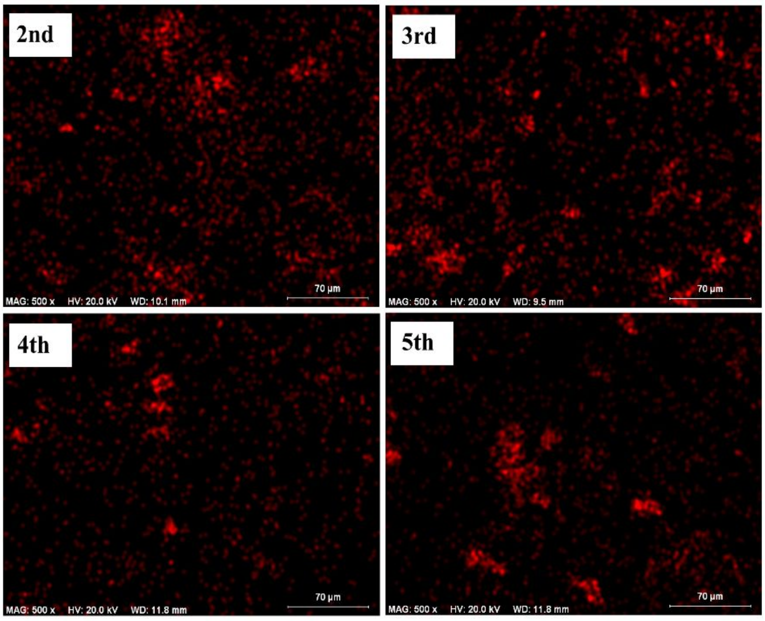


**Figure S2.** Structural stability of $FG_{1.0}$ ferrogels after repeated AMF exposure: SEM-EDS elemental mapping of Iron (Fe) distribution following the 2nd, 3rd, 4th, and 5th heating cycles.

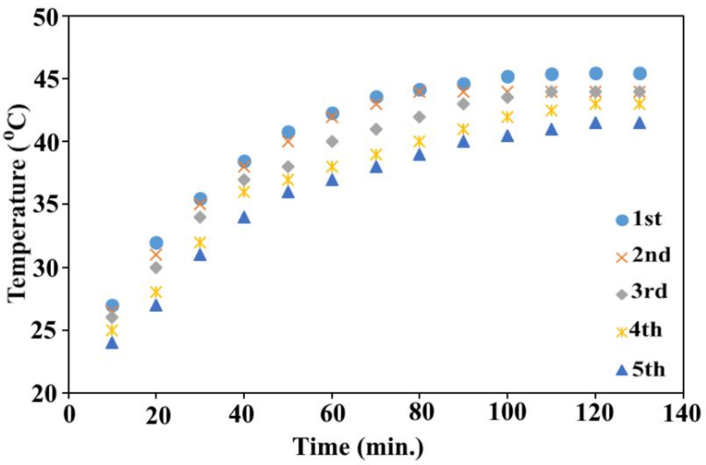


**Figure S3.** Magnetothermal stability and reusability of $FG_{1.0}$ ferrogels: Temperature-time profiles over five consecutive AMF heating cycles (1.25 mT) in 50 mL of deionized water.

**Table S1.** Different kinetic model equations for drug release.

| Kinetic models | Linear equations | Plots |
|---|---|---|
| *Zero-order* | $Q_t = Q_{eq} + K_0 t$ | $Q_t$ vs t |
| *First-order* | $\ln Q_t = \ln Q_0 - K_1.t$ | $-\ln Q_t$ vs t |
| *Higuchi* | $\ln Q_t = \ln K_H + 0.5\ln t$ | $\ln Q_t$ vs $0.5\ln t$ |
| *Korsmeyer–Peppas* | $\ln(Q_t/Q_{eq}) = \ln k_p + n.\ln t$ | $\ln Q_t/Q_{eq}$ vs $\ln t$ |

where; $K_0$, $K_1$ and $K_H$ are drug release parameters describing zero order con stant, first order constant and Higuchi constant, respectively. In all models, $Q_t$ parameter relates to the amount of drug dissolve data anytime. In Korsmeyer–Peppas model, $K_p$ is aconstant describing the drug-sample interaction and n is the release exponent describing the transport mechanism and $Q_t/Q_{eq}$ is the fraction of drug release data anytime.[67,68]

**Table S2.** Correlation coefficient ($R^2$) values of drug release kinetics of ferrogels according to different models under 1.25 mT.

| Sample | Zero-order release model | First-order release model | Higuchi model | Korsmeyer–Peppas model |
|---|---|---|---|---|
| $FG_{0.5}$ | 0.610 | 0.714 | 0.913 | 0.996 |
| $FG_{1.0}$ | 0.587 | 0.688 | 0.896 | 0.993 |
| $FG_{1.5}$ | 0.634 | 0.747 | 0.937 | 0.995 |
| $FG_{2.0}$ | 0.667 | 0.749 | 0.945 | 0.999 |

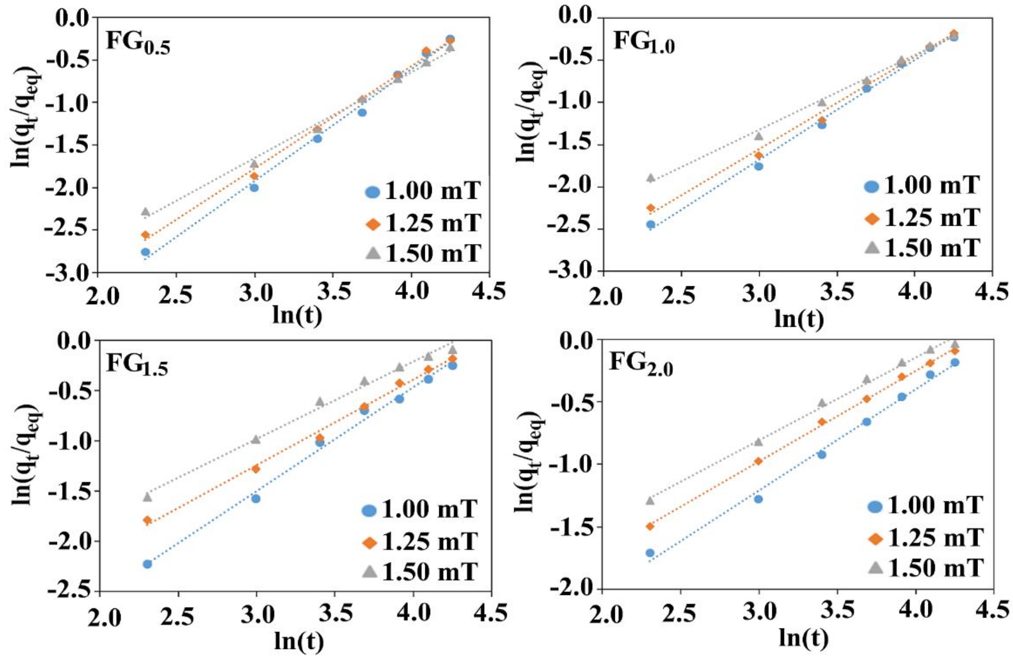


**Figure S4.** Korsmeyer–Peppas model fitting to ferrogel drug release kinetics

**Table S3.** Drug release characteristics of ferrogels for the Korsmeyer–Peppas model

| Sample | 1 mT | | | 1.25 mT | | | 1.50 mT | | |
|---|---|---|---|---|---|---|---|---|---|
| | $R^2$ | *K* | *n* | $R^2$ | *K* | *n* | $R^2$ | *K* | *n* |
| **$FG_{0.5}$** | 0.994 | 2.82 | 1.12 | 0.996 | 4.53 | 0.99 | 0.994 | 9.37 | 0.97 |
| **$FG_{1.0}$** | 0.995 | 5.39 | 0.99 | 0.993 | 7.72 | 0.98 | 0.996 | 18.61 | 0.84 |
| **$FG_{1.5}$** | 0.993 | 9.95 | 0.98 | 0.995 | 22.37 | 0.81 | 0.992 | 37.15 | 0.79 |
| **$FG_{2.0}$** | 0.994 | 26.24 | 0.76 | 0.999 | 42.47 | 0.75 | 0.995 | 61.11 | 0.68 |

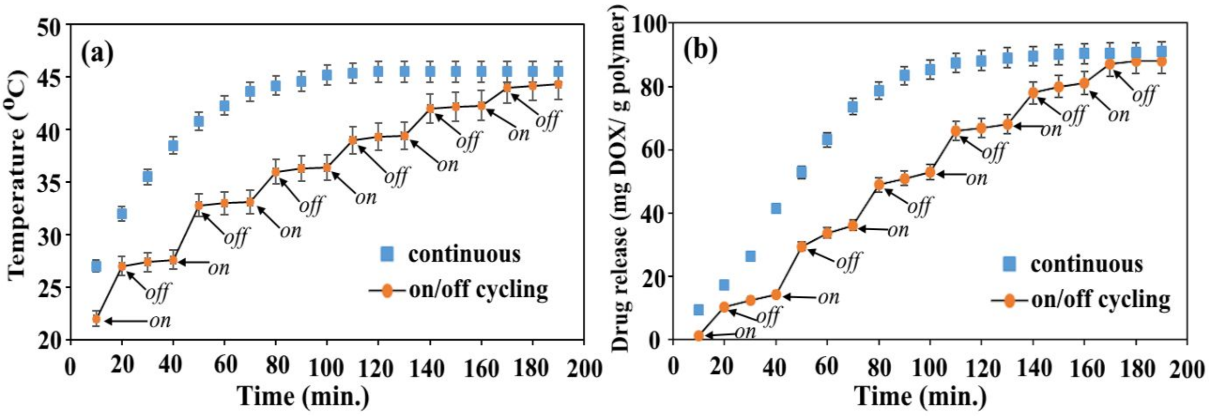


**Figure S5.** Real-time monitoring of the remote-controlled behavior of $FG_{1.0}$ ferrogels: (a) Temperature-time profiles and (b) cumulative DOX release kinetics under continuous and on/off cycling AMF exposure (1.25 mT, 360 kHz).

**References:**

(S1) Ilgın, P.; Ozay, H.; Ozay, O. A New Dual Stimuli Responsive Hydrogel: Modeling Approaches for the Prediction of Drug Loading and Release Profile. Eur. Polym. J. 2019, 113, 244–253.

(S2) Erikci, S.; van den Bergh, N.; Boehm, H. Kinetic and Mechanistic Release Studies on Hyaluronan Hydrogels for Their Potential Use as a pH-Responsive Drug Delivery Device. Gels 2024, 10, 123–135.
